# Modeling potential impact of COVID-19 pandemic on global electric vehicle supply chain

**DOI:** 10.1016/j.isci.2022.103903

**Published:** 2022-02-12

**Authors:** Xin Sun, Gang Liu, Han Hao, Zongwei Liu, Fuquan Zhao

**Affiliations:** 1 State Key Laboratory of Automotive Safety and Energy, Tsinghua University, Beijing 100084, China; 2 Tsinghua-Rio Tinto Joint Research Center for Resources Energy and Sustainable Development, Tsinghua University, Beijing 100084, China; 3 SDU Life Cycle Engineering, Department of Green Technology, University of Southern Denmark, 5230 Odense, Denmark; 4 Tsinghua Automotive Strategy Research Institute, Tsinghua University, Beijing 100084, China

**Keywords:** Electrochemical energy engineering, Energy transportation, Transportation engineering

## Abstract

The on-going COVID-19 pandemic and consequent lockdowns cast significant impacts on global economy in the short run. Their impact on stability of global electric vehicles (EVs) supply chain and thus our climate ambition in the long run, however, remains hitherto largely unexplored. We aim to address this gap based on an integrated model framework, including assessing supply risks of 17 selected core commodities throughout the EV supply chain and further applying the supply constraints to project future EV sales until 2030. Our model results under three pandemic development scenarios indicate that if the pandemic is effectively contained before 2024, the global EV industry will recover without fundamentally scathed and thus can maintain the same growth trend as in the no-pandemic scenario by 2030. We suggest that fiscal stimulus in the postpandemic era should be directed more toward upgrading the quality of battery products, rather than expanding the production capacity.

## Highlights

• Lithium is the most critical commodity for the electric vehicle industry
• Short-term COVID-19 pandemic will not cause constraints on the production side
• Long-term pandemic can seriously jeopardize electric vehicle market development
• Postpandemic policies should focus more on improving battery quality

## Graphical abstract

**Figure:**
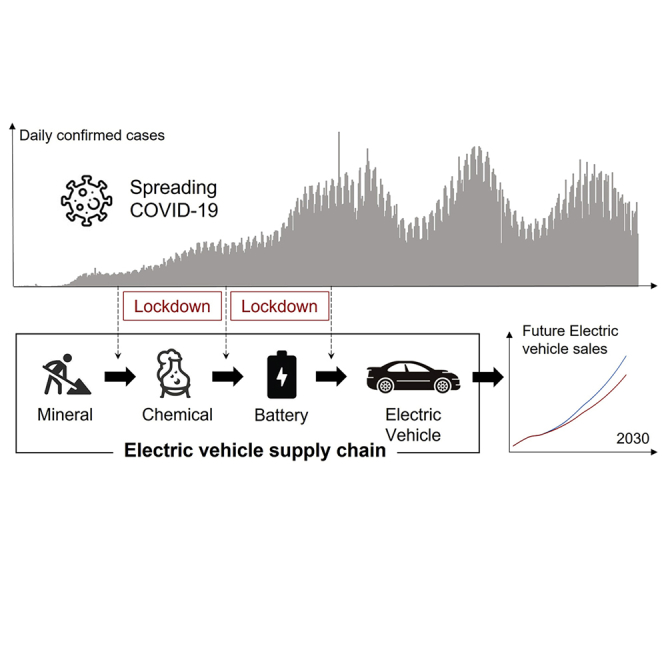

## Introduction

The COVID-19 pandemic has cumulatively caused 300 million confirmed cases worldwide, with three million deaths reported by January 7th, 2022 (Dong et al., 2020). Most countries have imposed “lockdown” measures to contain the spread of coronavirus when their domestic pandemic has reached a relatively severe level, including, e.g., mobility restriction, border closures, and plant shutdown. At the peak of the lockdown, economic activities around the world came to a near standstill (Diffenbaugh et al., 2020). The consequences caused by lockdown measures include not only the disruption of existing production but also the suspension of construction of new facilities. During a pandemic, the delay of production capacity expansion can be irreversible and thus affect the supply in the long run despite a possible gradual recovery of production and consumption to the prepandemic level with the lift of lockdown restrictions. For example, it is estimated that China’s newly installed capacity of battery production in 2020 would be 26 GWh lower than originally planned due to the pandemic restriction measures (Wood Mackenzie, 2020). Although vaccination is becoming widely available, there are still many uncertainties in future development of the pandemic (Jason, 2020; World Health Organization, 2020b). Thus, it is very possible that lockdown measures will be repeatedly imposed until the pandemic is fully contained (which already happened in some countries), causing suspensions of production and capacity building for several times.

Another crisis faced by humankind is the climate change, which persists longer and has a more extensive impact (Geng et al., 2018). To address the climate urgency, various countries have put forward ambitious emission reduction targets and corresponding development plans for electric vehicle (EV) (Crabtree, 2019; Hoekstra, 2019; Smriti, 2020; Wang et al., 2018). The necessary prerequisite for achieving these goals is a commensurate increase in the supply of relevant components and raw materials (Baars et al., 2020; Fu et al., 2020; IEA, 2021a; b; Watari et al., 2019). Facing the unclear COVID-19 pandemic development, it is vital to explore the impact of this major public emergency on stability of EV supply chain.

A number of studies have evaluated the impacts of the pandemic on human society from the perspectives of energy demand and associated environmental implications (Forster et al., 2020; He et al., 2020; Kikstra et al., 2021; Le Quéré et al., 2020; Shan et al., 2020), critical material supply stability (Ata et al., 2020; Roskill, 2020; Zhu et al., 2021), energy security (Gillingham et al., 2020; Marina et al., 2020; Ou et al., 2020; Ruan et al., 2020; Si et al., 2021), and economic losses (Guan et al., 2020). A few studies have explored the impact of the COVID-19 pandemic on EV industry. For example, McClone et al. (2021) studied the change on EV charging demand in the University of California, San Diego during the pandemic (McClone et al., 2021). Arribas-Ibar et al. (2021) studied the EV innovation ecosystem response to the pandemic (Arribas-Ibar et al., 2021). Wen et al. (2021) summarized the impacts of pandemic on China’s EV industry from the supply chain side and demand side for the year of 2020. They found that the COVID-19 pandemic has reduced the EV production and sales on the short term, but it also stimulated future EV demand (Wen et al., 2021).

**Figure 1:**
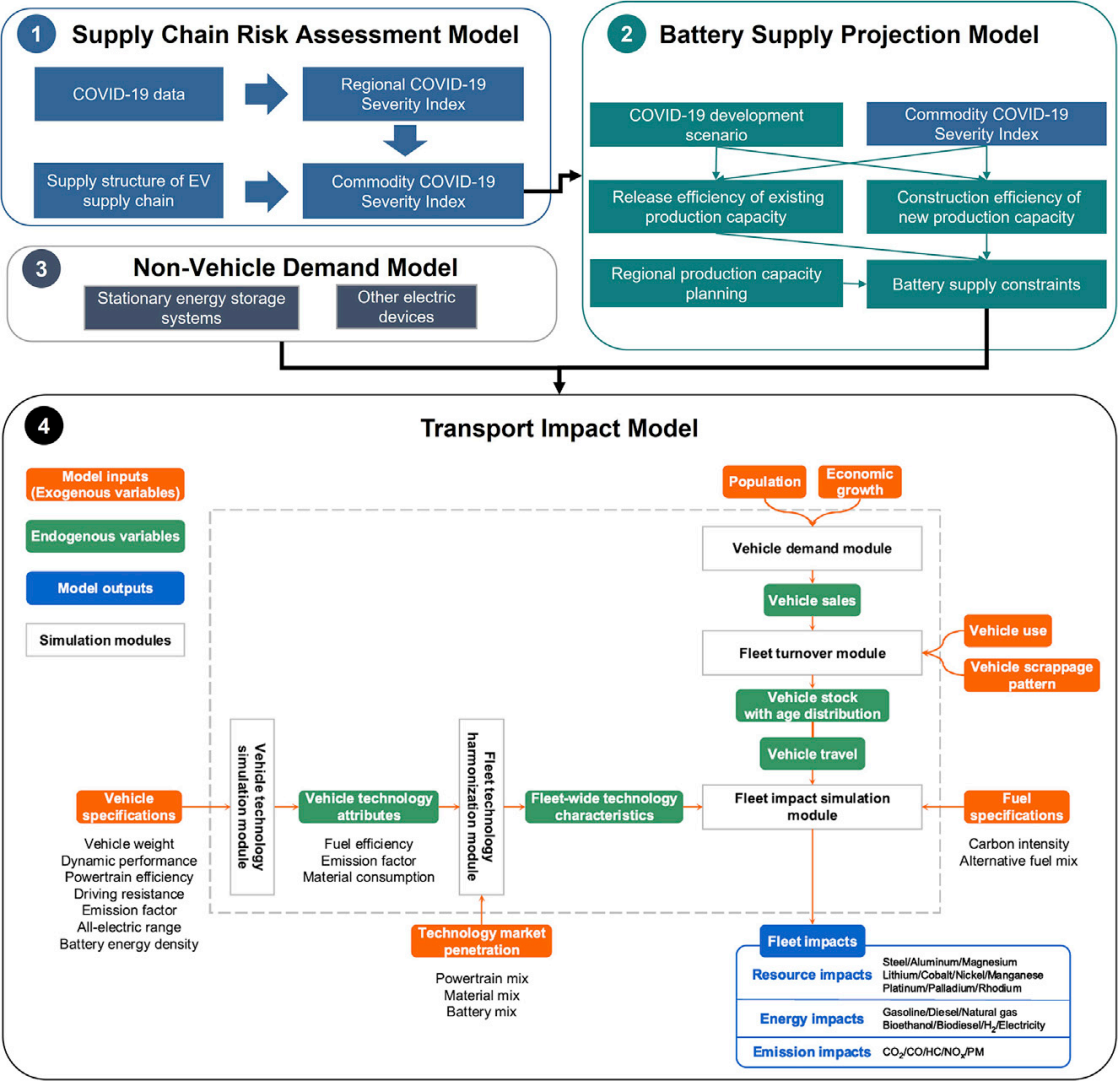
Integrated model structure for evaluating the potential impact of COVID-19 pandemic on global EV supply chain

Previous literature provided static evaluation for the existing impacts of the pandemic on EV industry. However, potential dynamic impact of lockdown measures on supply-demand balance of global EV industrial chain in the long run has hitherto not been fully investigated. To fill this gap, we developed an integrated model to explore whether the supply delays caused by the COVID-19 pandemic will be a bottleneck on the growth target of global EV market. The integrated model structure is shown in Figure 1. We first quantified the severity of the pandemic in 185 countries and regions to evaluate the probability of supply disruption of 17 core EV-related commodities throughout its supply chain. Based on these results, we further developed a bottom-up projection model to estimate available lithium-ion battery (LIB) production capacity under three pandemic development scenarios from 2020 to 2030. Such production constraints were then incorporated as input in the Transport Impact Model (Hao et al., 2019a, 2019b) to assess future EV sales.

Here we show that lithium mineral is the most critical raw material that will likely form restrictions on the EV industry due to the pandemic. If the pandemic could be gradually contained in the short term, the growth trajectory of EV market will not be affected by lockdown measures. Current excess production capacity of LIBs and relevant raw materials will provide a large buffer for supply disruption constraints on consumption growth. However, if the pandemic spread is not controlled in the short term, the development of EV industry could be seriously jeopardized before 2030. The results imply that the postpandemic recovery of EV industry should seek the window of opportunity created by this pandemic to upgrade the quality rather than the quantity of production capacity.

## Results

### Identification of risk sources in the EV supply chain

**Figure 2:**
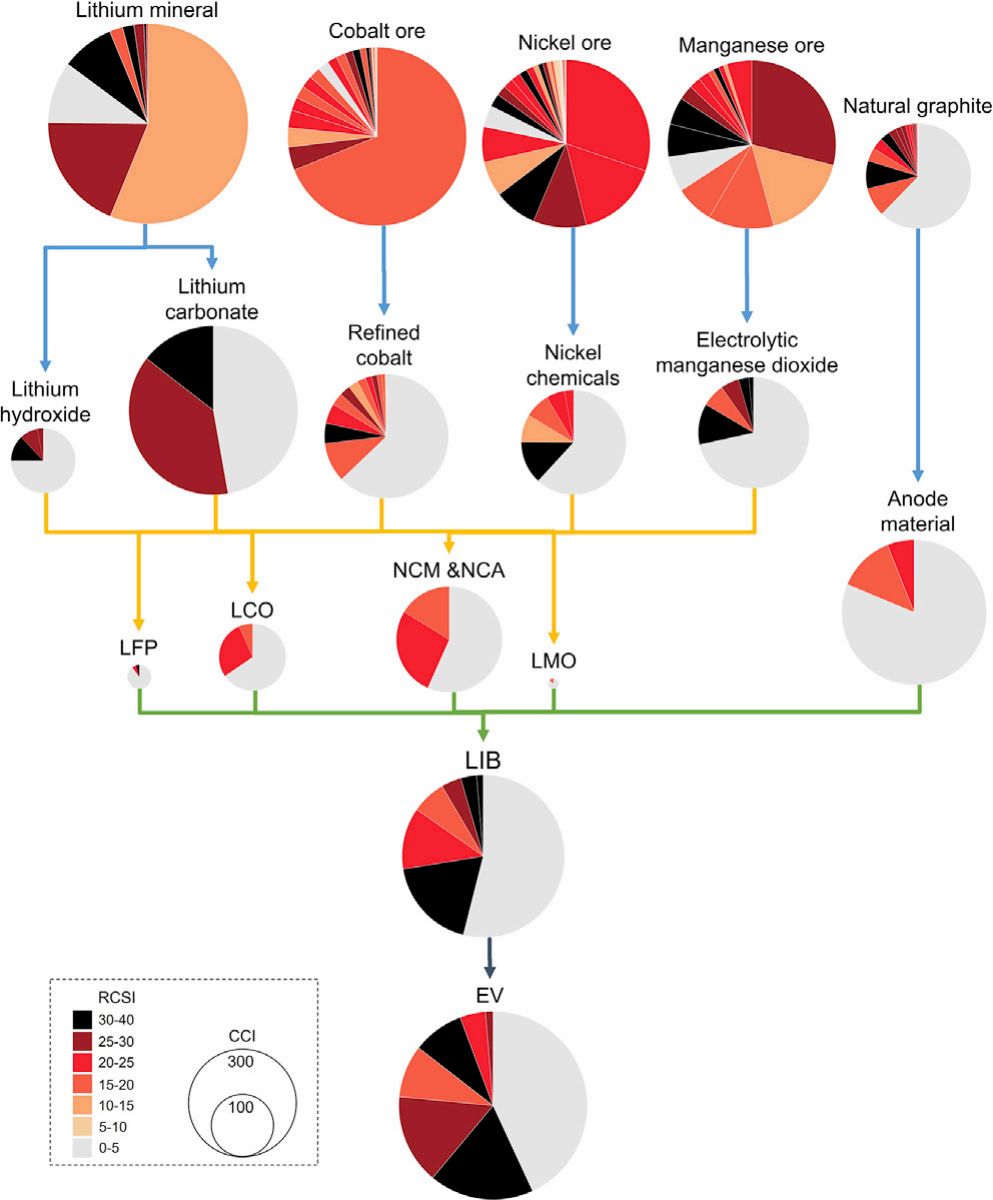
Severity of COVID-19 pandemic throughout the global EV supply chain LFP: lithium iron phosphate; LCO: lithium cobalt oxide; NCM & NCA: lithium nickel cobalt manganese oxide and lithium nickel cobalt aluminum oxide; LMO: lithium manganese oxide; LIB: lithium-ion battery; EV: electric vehicle; RCSI: regional COVID severity index; CCI: commodity criticality index. The pie chart corresponds to the supply structure of EV-related commodities. Each slice corresponds to proportion of each country’s supply. The colors of the slices correspond to the values of RCSI. The pie size is proportional to the value of CCI. The direction of the arrow is from the upstream commodity to the downstream commodity.

The current EV supply chain covers thousands of relevant components and raw materials (Bhuwalka et al., 2021). Considering the availability of data, their relative significance, and the operability of the model, we focused on the commodities related to the core component—LIB, which is the most valuable part that determines the performance of EV products (Crabtree, 2019; Olivetti et al., 2017). A total of 17 commodities were identified most relevant for the EV supply chain, from battery-related critical minerals to final products (Sun et al., 2017, 2018b, 2019a, 2019b, 2020). The distribution of supply of different commodities and the extent to which different countries are affected by the pandemic cause the diverse probability of supply disruption of various commodities. Based on the infection rate and growth rate of COVID-19 confirmed cases on the country level, we developed a Regional COVID-19 Severity Index (RCSI) to quantify the severity each country is affected by the pandemic. Further a Commodity Criticality Index (CCI) was proposed to reflect the potential risk embodied in the supply of each of the 17 commodities based on the corresponding supply structure by country (see Tables S4–S20), RCSI, and the impact of commodities on the EV industry. The detailed methodologies of calculating RCSI and CCI are presented in STAR Methods section. The final quantization results are shown in Figure 2 (see Tables S1–S3 for detailed values).

In 2019, a total of 63 countries and regions were linked to the EV supply chain. Among these countries, Turkey, the USA, Brazil, Argentina, and Spain were the top five countries with the highest RCSI (39, 36, 36, 34, and 32, respectively), whereas China (mainland), Taiwan, Australia, Madagascar, Burma, and Ghana were the five regions with the lowest RCSI (1, 10, 10, 13, and 14, respectively). These variations between countries reflect mainly the differences in their regional infection rate.

**Figure 3:**
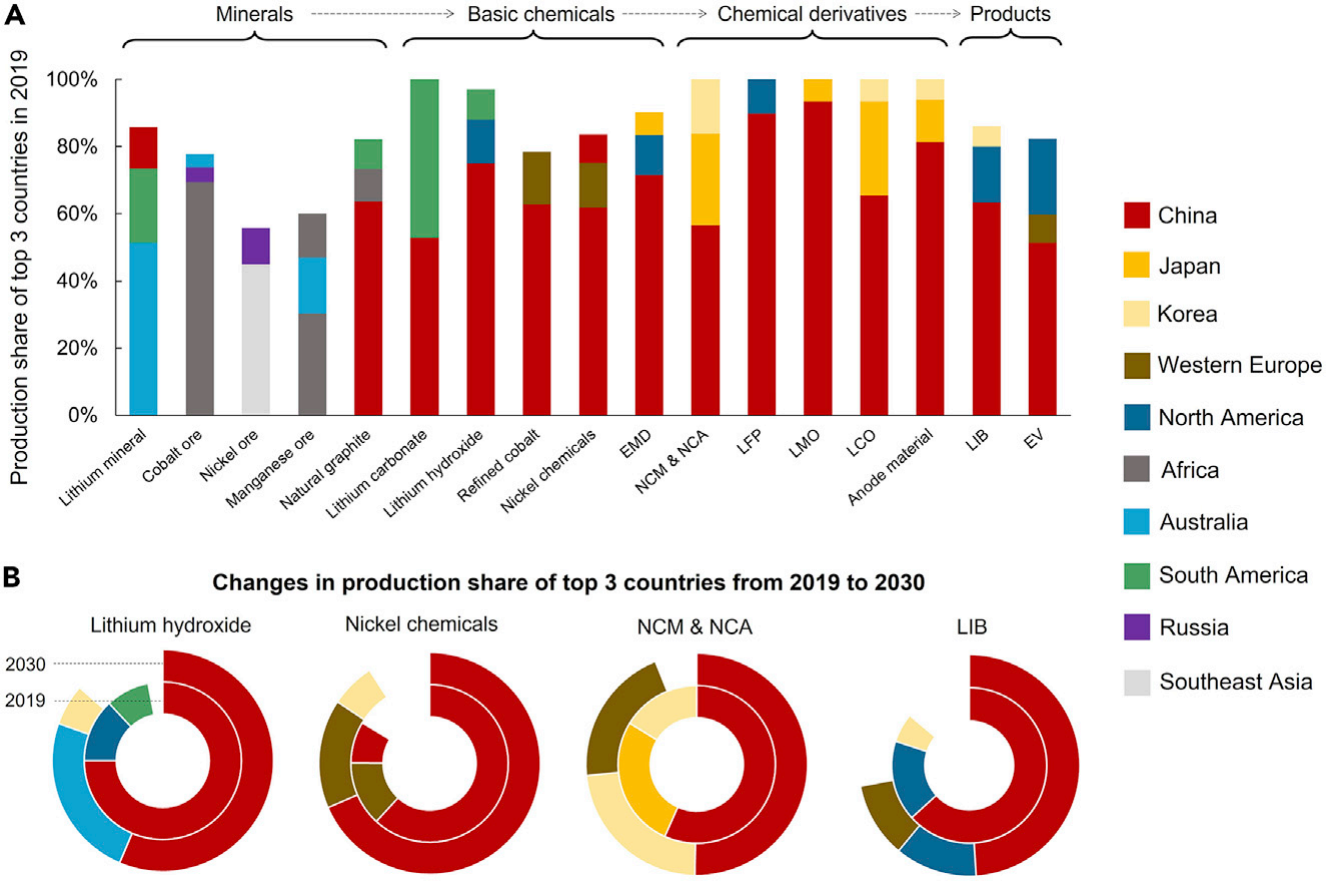
Top three suppliers in the EV supply chain for the year of 2019 and 2030 (A) The historical data for the proportion of the top three suppliers of LIB-related commodities in 2019. (B) The projected results of commodities of which there will be great changes in the production structure in 2030. EMD: electrolytic manganese dioxide. The inner circle of the torus represents the data for 2019, and the outer circle represents the data for 2030.

Supply of lithium mineral, EV, cobalt ore, nickel ore, and lithium carbonate were the greatest risk sources for EV industry during the pandemic, with the highest CCI of 1622, 1436, 1297, 1171, and 1159, respectively. The high CCI of lithium mineral was ascribed to the large production proportion of Chile and Argentina (with an RCSI of 28 and 33, respectively), as Figure 3A shows, and its irreplaceable role in automobile power battery industry. The USA, France, and Germany were the main sources for the high CCI of EV, with an RCSI of 36, 29, and 27, respectively. The CCIs of LIB cathode materials were relatively low: 5 for lithium manganese oxide (LMO); 23 for lithium iron phosphate (LFP); 181 for lithium cobalt oxide (LCO); and 454 for lithium nickel cobalt manganese oxide and lithium nickel cobalt aluminum oxide (NCM & NCA). The low CCI of these commodities benefited from the dominating positions of China, Japan, and Korea on supply (with an RCSI of 1, 17, and 19, respectively).

As the EV industry is still at the phase of rapid growth, the supply structure of EV-related commodities would change substantially in the future due to different investment strategies of various enterprises and countries. Therefore, the production data for 2019 may not accurately reflect the dynamic risks of EV supply chain in the next decade. We collected the information about production layout of relevant suppliers for the 17 selected commodities (see Tables S22–S25 for details). A significant change is then expected in the supply structure of lithium hydroxide, nickel chemical, NCM & NCA, and LIB by 2030 (see Figures 3B and S2–S7). Although China will remain as the largest supplier of these commodities, the second or third largest supplier will change. Taking these prospective changes in supply structure into account, the CCI of lithium mineral will remain higher than that of other commodities (Sun et al., 2020; USGS, 2021). As a result, lithium mineral was identified as the most critical source of supply constraint.

### Quantification of supply constraints from the pandemic

**Figure 4:**
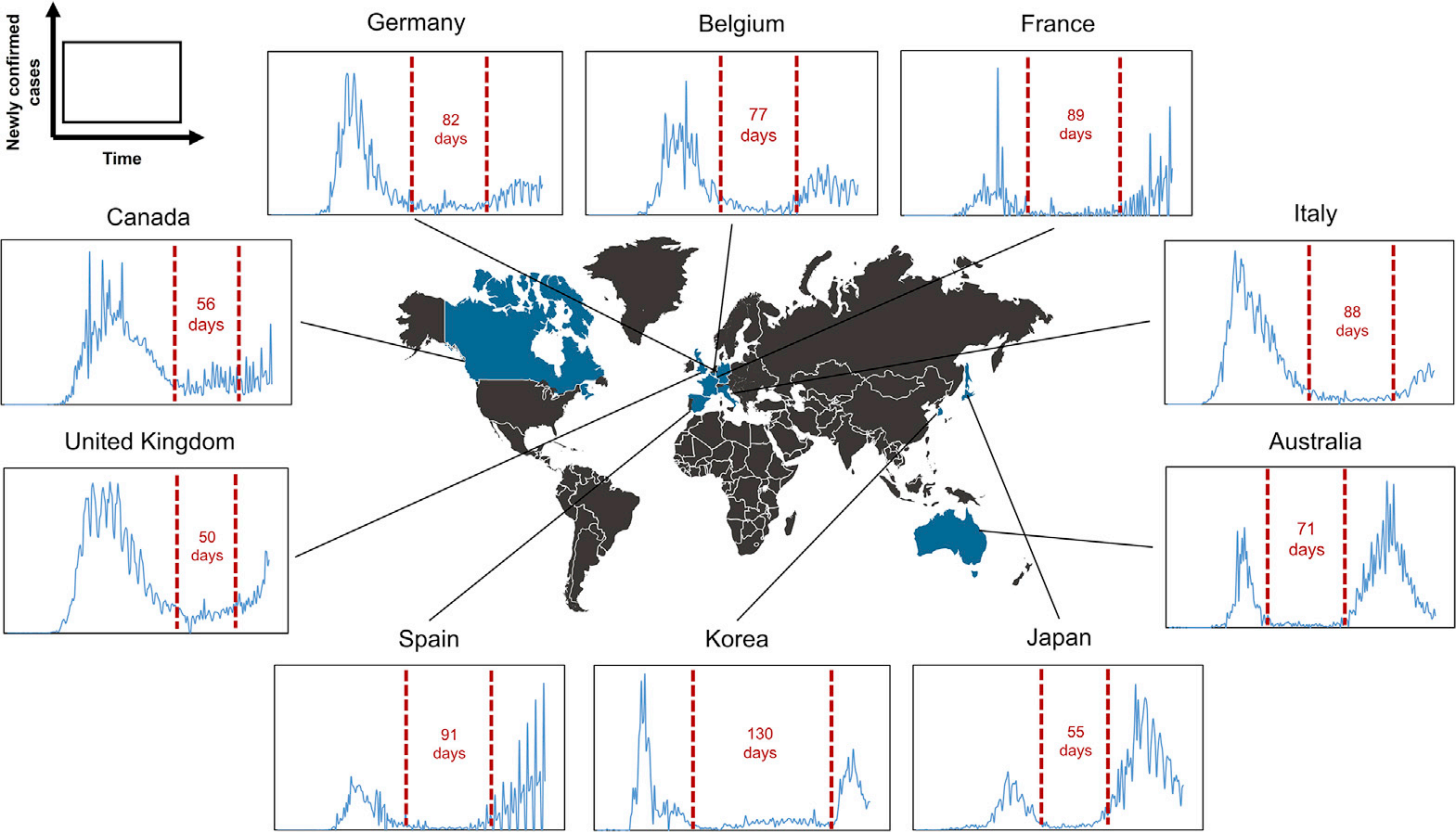
Time interval between the first and the second outbreak of COVID-19 in typical countries Blue lines show the newly confirmed cases. Red dotted lines show the end time of the first outbreak (when newly confirmed cases dropped below 10% of its first peak) and the beginning time of the second outbreak (when newly confirmed cases reached 10% of its second peak). Typical countries refer to the countries that have certain nucleic acid testing capabilities and have contained the first wave of pandemic but are suffering the second wave of pandemic.

The COVID-19 pandemic affects the supply-demand balance in multiple dimensions, including for example factory shutdown, logistics disruption, business failures, and purchasing power decline. In this study, quantification of the impacts of the pandemic on EV market begins on the supply side. As the information about long-run supply planning of lithium minerals is not available and lithium is currently essential for all EV battery technology routes, we mapped the probability of supply disruption of lithium minerals into LIB supply constraints and ignored the impacts of nonbattery applications of lithium. We developed the Battery Supply Projection Model based on the production capacity planning of LIB suppliers and two key parameters: (1) release efficiency of existing production capacity, quantifying the influence of the production disruption caused by the lockdown measures on the annual output of commodities; (2) construction efficiency of new production capacity, quantifying the influence of suspension of new plant construction on annual growth rate of production capacity. Model details are described in the STAR Methods section. In short, the underlying data to calculate these two parameters are the duration of each lockdown measure and the interval between the two waves of pandemic. The average duration of a single lockdown period is 64 days, according to a collation of information on relevant measures taken by countries in the first half of 2020 (Table S21). Based on the data of newly confirmed cases in typical countries (Dong et al., 2020), the average interval between two waves of COVID-19 pandemic is 79 days (Figures 4 and S1).

There is still high uncertainty about the future development of the COVID-19 pandemic. Therefore, we designed three scenarios to explore the possible consequences of different pandemic development pathways. The main difference between the various scenarios lies around the assumptions about the duration of the pandemic. Scenario assumptions and corresponding model parameters are shown in Table 1. The business-as-usual (BAU) scenario is the reference scenario used to provide the future EV market size by the year of 2030 based on the established climate targets with no impact of COVID-19 pandemic. The short-term recovery (STR) scenario is the conventional scenario in which the pandemic will be contained within a few years with the promotion of vaccines as World Health Organization (WHO) expected (World Health Organization, 2020a). The long-term coexistence (LTC) scenario is the extreme scenario in which the pandemic will not be contained within a decade.

**Table 1:** Set-ups of three pandemic scenarios

Scenario	Model assumptions	Model parameters	Notes
Business as usual (BAU)	Supposing that the COVID-19 pandemic did not happen. Establishment rate of EV production capacity could sustainably meet the growth of EV consumption. EV sales successfully reached the origin targets set by all countries.	Construction efficiency of new facilities: 100% from 2020 to 2030. Release efficiency of existing facilities: 100% from 2020 to 2030.	We assume that all current production capacity planning could be successfully reached.
Short-term recovery (STR)	Although some countries have halted the spread of the coronavirus, most have failed to do so. In most countries the number of confirmed cases rise again after relieving lockdown measures. Outbreaks bounce back frequently so that lockdown measures have to be reimposed for multiple times. The vaccine’s progress, however, is more optimistic. More than 20% of the global population each year can be immunized through successful marketing, mass production, and effective distribution of vaccines in 2021. By 2024, the coronavirus herd immunity will have been formed, making the pandemic situation disappeared in human society. Production and consumption activities have subsequently returned to the original trajectory.	Construction efficiency of new facilities: 82% in 2020; 87% in 2021; 91% in 2022; 96% in 2023; 100% from 2024 to 2030. Release efficiency of existing facilities: 86% in 2020; 89% in 2021; 93% in 2022; 96% in 2023; 100% from 2024 to 2030.	Based on the duration of each lockdown measure and the interval between the two waves of pandemic, we assume that lockdown measures will be taken two times per year. Final calculation results of construction efficiency and release efficiency are 82% and 86% in the initial year, respectively. We assume that the release efficiency and construction efficiency will increase linearly to 100% by 2024 because of promotion of vaccines.
Long-term coexistence (LTC)	Most countries have failed to bring pandemic under full control through governance measures. Worse still, because developed vaccines are not as safe, effective, and productive as expected, the vaccine fail to stop the spread of the virus until 2030. Over the next decade, the pandemic continues to spread. Many countries have to take lockdown measures for lots of times.	Construction efficiency of new facilities: 82% from 2020 to 2030. Release efficiency of existing facilities: 86% from 2020 to 2030.	We assume that construction efficiency and release efficiency will keep the same as they are in the initial year, respectively.

### Prospective EV market under three scenarios

The end-use applications of LIBs were divided into three sectors: EVs (including passenger vehicles, light-duty commercial vehicles, and heavy-duty commercial vehicles), stationary energy storage systems, and other electric devices (including communication equipment, portable computers, electric two/three wheelers, laptops, cameras, power tools, drones, and robots). We assumed that the EV sector would be the first to be affected by LIB supply constraints due to its large share in consumption, high technical requirements, and high substitutability (e.g., internal combustion engine vehicle could fully substitute LIB and EV’s roles in meeting mobility demand). The future consumptions of stationary energy storage systems and other electric devices were modeled consistently under these three scenarios. Then we can get the available LIB production capacity for EVs by subtracting the demand for other two sectors from the total production capacity. Further we applied the available LIB production capacity for EVs into our developed TIM as the constraint condition to calculate prospective sales of LIB-powered EVs.

**Figure 5:**
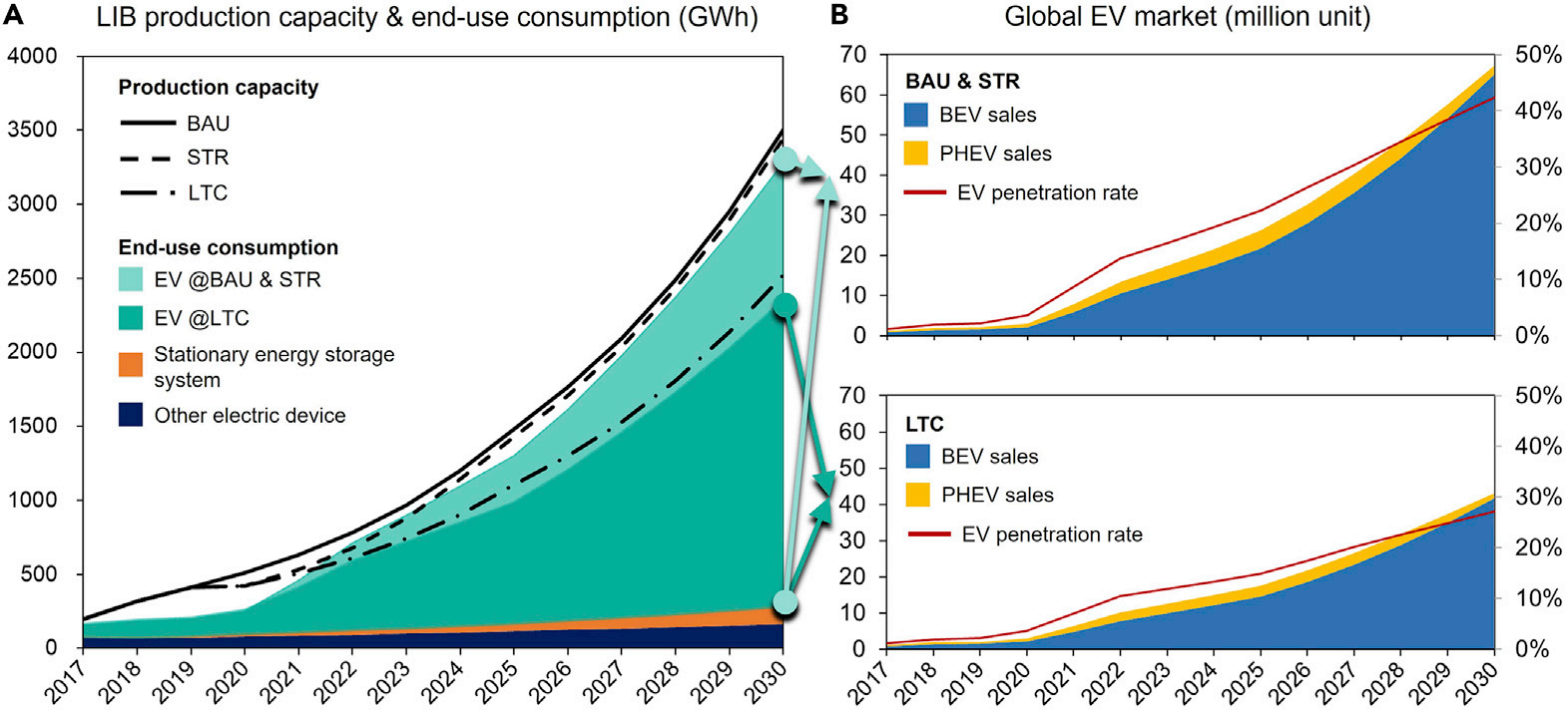
Projections of LIB production capacity, end-use LIB consumption, and corresponding EV sales under three pandemic development scenarios BEV: battery electric vehicle; PHEV: plug-in hybrid electric vehicle. (A) Lines correspond to the global LIB production capacity under the three scenarios. Stacking areas correspond to the end-use consumption of LIBs. The colors of four areas correspond to four end-use categories of LIBs. Cyan areas in different transparency correspond to the demand from EVs under three scenarios. Their lower boundaries are the same and part of their areas overlap. (B) Stacking area maps in different colors correspond to sales of EVs with different powertrain systems (left vertical axis). Red lines correspond to annual market penetration rate of EV sales (right vertical axis). Two subgraphs correspond to different scenarios.

Prospective production capacity and end-use consumption of LIBs are shown in Figure 5. On the supply side, the total LIB production capacity could reach 3505 GWh by 2030, steadily rising from 200 GWh in 2017 under the BAU scenario. Under the STR scenario, the LIB production capacity will first slightly increase to 421 GWh in 2020 from 411 GWh in 2019 and then rise steadily to 3449 GWh in 2030. Under the LTC scenario, the LIB production capacity will reach 2522 GWh in 2030, after the same trend before 2019 as that in the STR scenario.

On the demand side, LIB used in the nonvehicle applications will grow from 85 GWh in 2017 (stationary energy storage systems, 12; other electric devices, 73) to 290 GWh (stationary energy storage systems, 125; other electric devices, 165) in 2030, which are the same in all three scenarios due to our assumption. Consumption for LIBs in the EV sector will be the same under the BAU and STR scenarios, which will grow steadily to 2993 GWh in 2030 from 145 GWh in 2020. The penetration rate of EV will reach 42% by 2030 with annual sales of 2.1 million plug-in hybrid electric vehicles and 65.2 million battery electric vehicles. Under the LTC scenario, more restrictive production capacity will depress the consumption of LIBs in EV sector to 2051 GWh in 2030. The penetration rate of EVs will reach 27% by 2030 with sales of 1.4 million plug-in hybrid electric vehicles and 41.7 million battery electric vehicles per year. Detailed results of EV sales by vehicle type (passenger vehicles, light-duty commercial vehicles, and heavy-duty commercial vehicles) are shown in the Figures S15 and S16.

One significant finding of our model results is that the losses of LIB production capacity caused by COVID-19 pandemic will have little impact on the EV demand growth if the pandemic could be gradually contained before 2024. The underlying reason for this circumstance is the existing overcapacity for production of LIB in the past. From 2017 to 2019, the ratio of actual production to production capacity of LIBs kept decreasing from 75% to 51%. These excess production capacities form a large buffer for the shock of the pandemic on supply. Nevertheless, if the pandemic persists for long run, production capacity constraints will start to cast a devastating impact on the development of the EV industry.

## Discussion

### Uncertainty analysis

In the integrated model developed in this study, the model parameter with the highest uncertainty is the duration of strict lockdown measures (hereinafter referred to as lockdown duration). This parameter directly affects the construction efficiency of new facilities and release efficiency of existing facilities. We made a strong assumption for lockdown duration that this parameter would be consistent with the historical data during each wave of future outbreak, whereas factors such as the severity of future outbreaks and the speed and willingness of governments to respond could be very different from the past. Even for the same country, the lockdown duration is likely to change dramatically. In addition, there are great differences in the enforcement of pandemic prevention policies by national governments. We distinguish the impact of the lockdown duration on the opening rate at the country level based on the RCSI. But for countries with similar indexes, enforcement and focus of lockdown measures could vary greatly. The lockdown measures in some countries only stay at the level of controlling social distance in order to maintain the operation of the economy. Some other countries may suspend all economic activities in an effort to quickly contain the pandemic.

Another major source of uncertainty is the weight coefficient of the two parameters, infection rate and growth rate of COVID-19 confirmed cases, used to calculate RCSI. Due to the insufficient information supporting the weight set, we assumed that the two parameters contribute equally to the regional supply risk. The changes of weight coefficient may lead to the different relative size of the country’s RCSI value.

We consequently performed uncertainty analysis to test the impact of the uncertainty of lockdown duration and weight coefficient on the model output. The observed model output is the cumulative global EV sales from 2020 to 2030. The results of uncertainty analysis are shown in the Figures S16 and S17. Results indicate that model output is strictly negatively correlated with the lockdown duration. Under the STR scenario, with the lockdown duration parameter changing by −20%–20% of the original value, the cumulative global EV sales changes by 0.94% to −0.94%. Under the LTC scenario, with the lockdown duration parameter changing by −20%–20%, the cumulative global EV sales changes by 6.41% to −6.22%. The model output is sensitive to the input of lockdown duration, suggesting this parameter needs to be better considered in future studies. When we change the relative value of the weight coefficient of infection rate and growth rate linearly from 10: 90 to 90:10, the cumulative global EV sales change by −0.7%–1.2% under the STR scenario and by −1.6%–2.9% under the LTC scenario. Therefore, the model output is relatively not sensitive to the weight coefficient because the relative size of different countries' RCSI has not been fundamentally changed with the change of weight coefficient.

### Policy implications

This study investigated the potential impacts of COVID-19 pandemic on global EV industry using an integrated model framework that combines supply chain risk assessment, LIB supply constraint calculation, and EV market projection. Our model results could provide insights to inform policy for improving security of global EV supply chain.

Although the COVID-19 pandemic has caused a huge shock to the world economy, the pace of the energy transition has not been hindered, with countries strengthening the emission reduction regulations and adopting corresponding fiscal stimulus policies. Global total vehicle sales in 2020 reached 78.03 million units, with a 13% decrease from 2019. Global sales of EVs, however, rose by 43% to 3.2 million units. Our model results suggest that this development trajectory is promising to continue if the pandemic could be effectively controlled in the short-term because the quantity of production capacity is more likely not to be a constraint. However, it does not mean that the current overcapacity for LIB production is worth advocating; this is particularly true considering that the automotive power battery market presents an oversupply of low-grade batteries and inefficiency of high-grade batteries (Chen, 2018). The market performance of leading enterprises and small-sized enterprises shows a polarization. The global production capacity utilization rate of automotive power batteries in 2019 was only 38% (collation of the enterprise public information, see Table S20). CATL and LG Chem, the two largest LIB suppliers, had a capacity utilization rate of 51%. The next top eight companies had an average capacity utilization rate of 33%. The remaining LIB companies were running at a capacity utilization rate of 22%. According to our estimation, current scheduled production expansion of LIB suppliers is sufficient to meet the future demand of LIBs before 2030. Capacity utilization will be maintained over 90% by 2030. Therefore, the future stimulus and investment should be directed more toward upgrading the manufacturing level and product performance, rather than expanding the production capacity.

It should be noticed that the above conclusion is drawn in reference to current EV penetration expectation. The focus of this study is to explore the impact of different pandemic development scenarios on production capacity and then to see if they will cause constraints on the original target. However, the impacts of the pandemic will be cast not only to the supply side but also to the demand side. For example, many studies suggest that stimulus funding in the postpandemic era should be spent on green industries (Forster et al., 2020; Gillingham et al., 2020; Marina et al., 2020). In case such policy advice is followed, EV sector as a typical example of the green industries can be further stimulated. Under such a circumstance, the LIB production capacity might become a constraint, given more aggressive EV growth target. Further researches are needed on the impact of different investment policies in the postpandemic era on the energy system.

Our results also suggest that the performance of countries in the pandemic should be incorporated into considerations on capacity planning. China’s low RCSI and large production share are the major reasons for its construction efficiency and release efficiency to remain over 80% under the LTC scenario. During the COVID-19 pandemic, government policies and executive forces vary significantly from country to country (Andreas and Llewelyn, 2020). It took China three months to bring the pandemic almost completely under control, keeping the number of domestic new confirmed cases under 1000. In the USA, the pandemic has been continuously going on, with daily confirmed cases fluctuating from tens of thousands to over 1 million. These gaps in industrial chain stability should be considered by relevant stakeholders in their capacity planning. For instance, for investment of lithium mining projects, priority might be given to better performing countries such as Australia. The above suggestions are put forward from the perspective of reducing the impact of large-scale public security incidents on the industrial chain. However, there is generally an inevitable “trade-off” between risk mitigation and cost control (Sun et al., 2019a). In addition, constraints from environmental, social, and governance (ESG) regulations on business investment are increasingly mighty (Roskill, 2020). Site selection and layout of production capacity, of course, need a multidimensional comprehensive evaluation system.

Despite the current progress of the vaccination is optimistic, it is still necessary to normalize the prevention and control of the coronavirus. Preparing for extreme scenarios such as the LTC is highly essential. The corresponding measures to maintain the stability of supply should be laid out in advance, such as establishing regular material inventory and maintaining the promotion of recycling (Ali et al., 2017; Harper et al., 2019). Our results highlight that lithium mineral is the most worth-noticing commodity for EV supply chain. These resource strategies can be further explored based on our integrated model as well.

### Limitations of the study

There are some limitations and uncertainties in our model structure and results. First, the pandemic data used in this study are adopted from public information. Because of inadequate testing, statistical biases, and delay in information disclosure, the actual number of infections may be different from the number recorded. In addition, as the pandemic was still under development when the study was finished, the final quantitative results of the study do not fully reflect the severity of the pandemic in each country.

Second, in the prediction of future LIB production and consumption, due to the deviation of model structure, model assumptions, and basic data, the results may be different from the actual situation. Future potential changes in energy technology and policy might remodel the EV supply chain significantly. The enterprise capacity planning will also keep changing either because the original capacity planning fails to reach the target or because the market performance changes. Model output of future EV market balance could be adjusted with updated information.

Third, this study only considered the risks transmitted from the LIB supply chain on EV industry. However, the supply disruption of other upstream commodities is also possible due to the spread of coronavirus. The supply of automotive-grade chips, for example, is already in serious trouble, and such situation is likely to continue until 2023 (Accenture, 2021). Because vehicle manufacturers tend to prioritize the limited supply of chips for the EV line, the impact of chip shortage has been so far largely confined to the fabrication of internal combustion engine vehicles. Whether the impact will extend to EVs is still uncertain. The supply chain of chips and other core components could be included in the model framework of further studies.

## STAR★Methods

### Key resources table

**Table:** 

REAGENT or RESOURCE	SOURCE	IDENTIFIER
**Deposited data**		

Database: COVID-19 Data Repository	(Dong et al., 2020)	https://github.com/CSSEGISandData/COVID-19
Supply distribution data of 17 EV-related commodities	This paper	N/A
Production capacity plan of 17 EV-related commodities	This paper	N/A

**Other**		

Transport Impact Model	(Hao et al., 2019a)	N/A

### Resource availability

#### Lead contact

Further information and requests for resources should be directed to and will be fulfilled by the lead contact, Han Hao (hao@tsinghua.edu.cn).

#### Materials availability

This study did not generate new unique reagents.

### Method details

The integrated model is consisted of four sub-model: (1) Supply Chain Risk Assessment Model; (2) Battery Supply Projection Model; (3) Non Vehicle Demand Model; (4) Transport Impact Model. The functions, specific composition and calculation logic of each sub-model are described as follows.

#### Supply chain risk assessment model

The Supply Chain Risk Assessment Model is used to quantify the possibility of supply disruption in the EV supply chain due to COVID-19 pandemic. The EV supply chain is consisted of over a thousand types of commodities. Considering the importance of these commodities to the performance of EVs and the availability of data (Sun et al., 2021), 17 key commodities were studied: lithium mineral, cobalt ore, nickel ore, manganese ore, natural graphite, anode material, lithium carbonate, lithium hydroxide, refined cobalt, nickel chemicals, electrolytic manganese dioxide, LFP, LCO, NCM & NCA, LMO, LIB, and EV.

We used the RCSI to quantify the severity of each country affected by the COVID-19 pandemic, as Equation 1.

(Equation 1) $$RCSI_{i}=50*\frac{IR_{i}-IR_{\text{min}}}{IR_{\text{max}}-IR_{\text{min}}}+50*\frac{GR_{i}-GR_{\text{min}}}{GR_{\text{max}}-GR_{\text{min}}}\;$$

Where,

*RCSI*_*i*_ is the RCSI of country *i*;
*IR*_*i*_ is the infection rate of COVID-19 in country *i* (the ratio of the cumulative confirmed cases to the country’s total population);
*IR*_*min*_ is minimum infection rate among all countries;
*IR*_*max*_ is maximal infection rate among all countries;
*GR*_*i*_ is the average daily growth rate of the cumulative number of confirmed cases;
*GR*_*min*_ is minimum average daily growth rate among all countries;
*GR*_*max*_ is maximal average daily growth rate among all countries.

RCSI is consisted of two sub-indexes: (1) infection rate and (2) average daily growth rate of cumulative COVID-19 confirmed cases at the country level. The infection rate of COVID-19 confirmed cases reflects the current status of regional pandemic diffusion. As the coronavirus is still spreading in many countries, the average daily growth rate of cumulative COVID-19 confirmed cases is used to reflect the future regional pandemic diffusion potential. The two sub-indexes are normalized respectively and then aggregated to calculate the RCSI. Here we assumed that the contribution of the two parameters is equal to the final pandemic severity and set the weight coefficients of the two parameters to the same size.

Further the CCI was calculated to quantify the possibility of supply constraints on EV market of each kind of commodities, as Equation 2.

(Equation 2) $$CCI_{j}=\sum \left(\frac{PO_{i}}{\sum PO_{i}}*RCSI_{i}\right)*I_{j}\;$$

Where,

*CCI*_*j*_ is the *CCI* of commodity *j*;
*PO*_*i*_ is the production of commodity *j* of country *i*.
*I*_*j*_ is the impact of commodity *j* on EV industry.

The commodity supply structure and the *RCSI* are used to calculate the probability of supply disruption of the commodity due to the pandemic. The more severe the pandemic is in the major producers of a commodity, the more likely the commodity’s supply will be disrupted. The impact of commodities on EV industry is quantified by proportion of EV sector associated with the commodity’s total demand. For example, all EVs are powered by LIBs, thus the impact index of LIB is 100%. 25% of LIBs use LCO as the cathode material, thus the impact index of LCO is 25%. Detail explanations and results of RCSI, impact of various commodities on EV industry, and *CCI* are shown in Tables S1–S3.

The COVID-19 data comes from the Center for Systems Science and Engineering at Johns Hopkins University (Dong et al., 2020). The temporal boundary runs until December 31st, 2021. The production data breakdown by country of various commodities is adopted from relevant databases and reports. The temporal boundary is the year of 2019. The reliability of the data sources has been tested in a series of our previous peer-review studies (Sun et al., 2017, 2018b, 2019a, 2019b, 2020). Detail data information and corresponding sources are provided in the Tables S4–S20.

#### Battery Supply Projection Model

The Battery Supply Projection model is used to estimate the future LIB production capacity, which is the maximum amount of LIBs that can be produced. It is modeled based on the expected growth rate of production capacity, release efficiency of existing production capacity, and construction efficiency of new production capacity, which is calculated as Equation 3. Time step of the model is one year.

(Equation 3) $$PC_{t}=RE_{t}*\left(PC_{t-1}+CE_{t}*PC_{t0}*{\left(EGR+1\right)}^{t-t0-1}*EGR\right)\;$$

Where,

*PC*_*t*_ is the production capacity of LIBs in year *t* (kWh);
*RE*_*t*_ is the release efficiency of existing production capacity in year *t*;
*CE*_*t*_ is the construction efficiency of new production capacity in year *t*;
*t0* is initial time of model input, which is set to be 2019;
*EGR* is the expected growth rate of production capacity of LIBs.

Expected growth rate of production capacity is the growth rate under the BAU scenario. We assume that the growth of production capacity is not affected by the COVID-19 pandemic in this scenario. We collected the information about announced capacity planning of LIB suppliers (Table S22). The capacity planning is divided into two phases: capacity to be achieved from 2020 to 2025 and that from 2025 to 2030, respectively. The compound annual growth rate (CAGR) calculated by the capacity in the final year and in the initial year of each phase is the expected growth rate as Equations 4 and s5.

(Equation 4) $$EGR_{1}={\left(\frac{\sum_{i}PPC_{i,\;2025}}{\sum_{i}PC_{i,2019}}\right)}^{\frac{1}{6}}-1=23.9\%\;$$

(Equation 5) $$EGR_{2}={\left(\frac{\sum_{i}PPC_{i,2030}}{\sum_{i}PPC_{i,2025}}\right)}^{\frac{1}{5}}-1=18.7\%\;$$

Where,

*EGR*_1_ is the expected growth rate in phase 1 (2020–2025);
*EGR*_2_ is the expected growth rate in phase 2 (2025–2030);
*PC*_*i*_,_2019_ is production capacity of LIBs of company *i* in 2019 (GWh);
*PPC*_2025_ is the planned production capacity of LIBs of company *i* by 2025 (GWh);
*PPC*_2030_ is the planned production capacity of LIBs of company *i* by 2030 (GWh).

Release efficiency of existing production capacity is estimated based on the current pandemic data and information about lockdown measures of each country. The average duration of a single lockdown period is 64 days (Table S21). The average interval between two waves of COVID-19 pandemic is 79 days. Thus the efficacy duration of lockdown measures is 143 days (64 + 79). The efficacy duration is more than 1/3 of one year but less than 1/2 of one year. Thus we assume that lockdown measures will be taken two times per year. Then we can get that the total duration of the lockdown measure per year is 128 days (64∗2).

Due to the difference of national governance framework, the operation situation of factories in different countries will be very discriminative during the period when the lockdown measures are taken. However, it is difficult to obtain the specific information of each country, and there is great uncertainty about whether the policy will remain the same after the outbreak of the pandemic again in the future. Here we use the RCSI to estimate the operation rate of each country during the lockdown period, which is assumed to be: 100% (RCSI: 0–10); 80% (RCSI: 10–15); 60% (RCSI: 15–20); 40% (RCSI: 20–25); 20% (RCSI: 25–30); 0% (RCSI>30). As stated in the results section, lithium mineral is the most critical commodity for LIB supply. Based on the current supply structure of lithium mineral (Table S4), only 59% of its production can be guaranteed, meaning that 59% of LIB production capacity can be released when lockdown measure is taken. Finally we can get that annual release efficiency of existing production capacity when the pandemic continues to spread is ((365–128) + 128∗59%)/365 = 86%. According to the WHO’s COVID-19 Vaccine Implementation Plan, 2 billion vaccines will be provided per year starting in 2021 (World Health Organization, 2020a). Considering the time required for distribution and injection, global herd immunity is expected to be achieved by 2024. Thus we assume that, under the STR scenario, the release efficiency of existing production capacity will increase linearly to 100% by 2024 (Table 1).

The calculation of construction efficiency of new production capacity is similar to that of release efficiency of existing production capacity. The difference is the operation rate of each country during the lockdown period, which is assumed to be: 100% (RCSI: 0–5); 50% (RCSI: 5–10); 0% (RCSI>10). The reason for this setting is that construction requires far more manpower than production activities. Based on the schedule of worldwide LIB production capacity layout (Table S20 and Figure S2), we can get the construction efficiency of new production capacity when the pandemic continues to spread is ((365–128) +128∗49%)/365 = 82%. Similarly, under the STR scenario, the construction efficiency of new production capacity will increase linearly to 100% by 2024 (Table 1).

#### Non-vehicle demand model

Except for EVs, the LIB applications can be divided into two categories: stationary energy storage system, and other electric device. The LIB demand in these two sectors are modeled independently.

The estimation of future LIB demand in stationary energy storage systems is based on the report of Lux Research (Lux Research, 2020). This report predicts that the LIBs used in stationary energy storage systems will reach 223 GWh by the year of 2035, from 15 GWh in 2019. Based on this information, we can get a CAGR of 18% from 2019 to 2035. Then we use this parameter to estimate the LIB demand in stationary energy storage systems from 2020 to 2030 with the exponential growth model, as Equation 6.

(Equation 6) $$LC_{SESS,t}=LC_{SESS,2019}*1.18^{t-2019}\;$$

Where,

*LC*_*SESS*_,_*t*_ is the LIB consumption in stationary energy storage systems in year *t* (GWh).

Other electric devices include cell phones, wireless headsets, smart watches, cameras, notebook computers, tablets, robots, drones, power tools, etc. Currently the consumer electronics is the major sector of LIBs used in other electric devices. From 2016 to now, sales of mobile phones and computers have been nearly unchanged. Future growth of LIBs used in other electric devices will come from increased demand for electricity from 5G applications and sales of other devices. We have predicted this demand in our previous published study (Sun et al., 2018a). In that study, we estimated that the LIB demand in other electric devices would reach the maximum amount of 280 GWh in 2050. Then based on this value and historical data from 2000 to 2016, we used the logistics model to estimate the annual consumption of LIBs in other electric devices. In this work, this model is modified using historical data from 2017 to 2019 as Equation 7.

(Equation 7) $$LC_{OED,t}=\frac{280}{\left(1+e^{\left(-0.1173*t+238.48\right)}\right)}+9.13\;$$

Where,

*LC_OED_* is the LIB consumption in other electric devices in year *t* (GWh).

#### Transport Impact Model

The LIB consumption in the EV sector is modeled based on the Transport Impact Model and output of the Battery Supply Projection Model. Vehicle types considered in the model include passenger vehicle, light-duty commercial vehicle, heavy-duty commercial vehicle. Transport Impact Model is a technology-rich, country-level, flow-driven approach model, developed by China Automotive Energy Research Center of Tsinghua University (Hao et al., 2019a, 2019b). The LIB consumption is the product of four factors: vehicle sales, EV market penetration, battery capacity per EV, and battery supply constraints as Equation 8. Time step of the model is one year.

(Equation 8) $$LC_{LDV,t}=\sum_{i}SA_{i,t}*MP_{t}*BC_{i,t}$$

Where,

*LC*_*LDV*,*t*_ is the LIB consumption in EVs in year *t* (GWh);
*SA*_*i*,*t*_ is the vehicle sales in year *t* in country *i* (unit);
*MP*_*t*_ is the EV market penetration in year *t*;
*BC*_*i*,*t*_ is the battery capacity per EV in year *t* in country *i* (kWh/unit).

The total vehicle sales is the product of country-level economy and population development forecast (see Figure S8). The baseline of regional EV market penetration rate is determined based on the vehicle electrification targets set by various countries (IEA, 2021a). In addition, the EV market penetration is the connector between our Battery Supply Projection Model and Transport Impact Model. This variable is adjusted to maximize the output of Transport Impact Model under the constraint of the result output of Battery Supply Projection Model as Equation 9. Figures S9–S12 show the detail country-level EV penetration projection results.

(Equation 9) $$MP_{t}=\max\left\{MP_{t}|\int_{t-2}^{t}LC_{LDV,t}<\int_{t-2}^{t}\left(PC_{t}-LC_{HDV,t}-LC_{SESS,t}-LC_{OED,t}\right)\right\}\;$$

The battery capacity per EV is obtained by solving the non-linear equations, as Equations 10 and 11. On one hand, the battery capacity determines the total energy that can be used for driving; On the other hand, the battery capacity itself affects the battery weight, which further affects the energy consumption rate of EVs. A larger battery capacity does not yield a proportionally larger electric range due to the increase in battery weight.

(Equation 10) $$ER_{t,i}=\frac{BC_{t,i}*\gamma }{EC_{t,i}}\;$$

(Equation 11) $$EC_{t,i}=\alpha *RF_{t,i}*\frac{{\left(BC_{t,i}*ED_{t,i}+CW_{t,i}\right)}^{\beta }}{PE_{t,i}}\;$$

Where,

*ER*_*t*,*i*_ is the electric range of vehicle sold in year *t*, in country *i* (km);
$EC_{t,i}$ is the energy consumption rate of vehicle sold in year *t*, in country *i* (MJ/km), which is the function of vehicle weight, aero and rolling resistance, and vehicle powertrain efficiency;
*RF*_*t*,*i*_ is the aero and rolling resistance factor of vehicle sold in year *t*, in country *i*;
*ED*_*t*,*i*_ is the battery energy density of vehicle sold in year *t*, in country *i* (kg/MJ);
*CW*_*t*,*i*_ is the vehicle curb weight (excluding battery weight) of vehicle sold in year *t*, in country *i* (kg);
*PE*_*t*,*i*_ is the powertrain energy efficiency of vehicle sold in year *t*, in country *i* (%);
αandβare the characteristics parameters, which reflect the rationale of vehicle energy consumption;
γis the percentage of battery energy that can be actually used out of the total battery capacity (%).

The key input data, including country-level EV sales, EV electric range, and battery technology evolvement in four categories of powertrain systems and three categories of vehicle models, are shown in the Figures S13–S16.

## Data Availability

• All underlying data used in this paper is available in the main text or the supplementary information or its sources have been clearly stated. • This paper does not report original code, which is available for academic purposes from the lead contact upon reasonable request. • Any additional information required to reanalyze the data reported in this paper are available from the lead contact upon request.
