# Supplementary material for: Modeling potential impact of COVID-19 pandemic on global electric vehicle supply chain
Source: iScience. 2022 Feb 12;25(3):103903. doi: 10.1016/j.isci.2022.103903 (PMC8837477; doi:10.1016/j.isci.2022.103903)
Supplement: Document S1. Figures S1–S18 and Tables S1–S25 [file mmc1.pdf]

iScience, Volume *25*

Supplemental information

Modeling potential impact of COVID-19 pandemic on global electric vehicle supply chain

Xin Sun, Gang Liu, Han Hao, Zongwei Liu, and Fuquan Zhao

Table S1. Regional COVID-19 Severity Index, Related to Figure 2.

Rank	Country	Regional COVID-19 Severity Index	Rank	Country	Regional COVID-19 Severity Index
1	West Bank and Gaza	64.11	94	Guyana	20.27
2	Bahrain	61.92	95	Bosnia and Herzegovina	19.75
3	Andorra	53.57	96	Grenada	19.54
4	Tanzania	44.01	97	Rwanda	19.19
5	Turkey	38.51	98	Cote d'Ivoire	19.15
6	Qatar	37.63	99	Oman	19.14
7	Kuwait	37.52	100	Korea, South	19.13
8	USA	36.17	101	United Arab Emirates	19.12
9	Brazil	36.02	102	Liechtenstein	19.11
10	Switzerland	35.58	103	Sri Lanka	19.07
11	Netherlands	35.48	104	Trinidad and Tobago	18.95
12	Panama	35.04	105	Nepal	18.89
13	Argentina	33.54	106	Kenya	18.71
14	Italy	33.50	107	Honduras	18.67
15	Jordan	33.44	108	Jamaica	18.66
16	Montenegro	32.76	109	Azerbaijan	18.37
17	Spain	32.47	110	Zambia	18.22
18	Colombia	32.15	111	Bangladesh	18.21
19	Peru	31.86	112	Eswatini	18.09
20	Serbia	31.74	113	Lesotho	18.02
21	Poland	31.31	114	Zimbabwe	17.69
22	Georgia	31.09	115	Kyrgyzstan	17.60
23	United Kingdom	30.91	116	Algeria	17.52
24	Israel	30.80	117	Fiji	17.48
25	India	30.57	118	Ecuador	17.26
26	Belgium	30.35	119	Japan	17.25
27	San Marino	30.14	120	Finland	17.24
28	Czechia	29.92	121	South Sudan	17.23
29	Estonia	29.39	122	El Salvador	17.22
30	France	29.27	123	Mozambique	17.04
31	Costa Rica	29.18	124	Cambodia	17.00
32	Sweden	29.05	125	Namibia	16.83
33	Iran	28.93	126	Nigeria	16.78
34	Slovenia	28.29	127	Congo (Brazzaville)	16.75
35	Belarus	28.12	128	Syria	16.63

36	Chile	28.05	129	Yemen	16.47
37	Mongolia	28.02	130	Bahamas	16.40
38	Ukraine	27.66	131	Cameroon	16.34
39	Germany	27.34	132	Thailand	16.30
40	South Africa	27.17	133	Vietnam	16.23
41	Iraq	27.11	134	Malta	16.06
42	Portugal	27.02	135	Saint Lucia	15.98
43	Lithuania	26.80	136	Monaco	15.70
44	Ireland	26.57	137	Belize	15.68
45	Mexico	26.23	138	Equatorial Guinea	15.58
46	Armenia	26.16	139	Sao Tome and Principe	15.57
47	Uganda	26.08	140	Senegal	15.49
48	Greece	25.79	141	Djibouti	15.37
49	Nicaragua	25.77	142	Congo (Kinshasa)	15.24
50	Brunei	25.73	143	Burkina Faso	14.91
51	Romania	25.71	144	Gabon	14.88
52	Denmark	25.43	145	Comoros	14.84
53	Paraguay	25.38	146	Iceland	14.68
54	Seychelles	25.33	147	Singapore	14.63
55	Russia	25.19	148	Central African Republic	14.43
56	Luxembourg	25.18	149	Dominica	14.32
57	Croatia	25.11	150	Benin	14.27
58	Lebanon	25.02	151	Papua New Guinea	13.95
59	Slovakia	24.78	152	Ghana	13.95
60	Norway	24.64	153	Guinea	13.83
61	Uruguay	24.33	154	Mauritania	13.71
62	Austria	24.24	155	Burma	13.69
63	Saudi Arabia	24.11	156	Sudan	13.52
64	Moldova	24.07	157	Angola	13.41
65	Tunisia	23.92	158	Timor-Leste	13.36
66	North Macedonia	23.91	159	Madagascar	12.95
67	Hungary	23.32	160	Somalia	12.91
68	Indonesia	23.18	161	Antigua and Barbuda	12.73
69	Libya	23.07	162	Saint Kitts and Nevis	12.42
70	Cabo Verde	22.81	163	Eritrea	12.40
71	Latvia	22.75	164	Guinea-Bissau	12.25
72	Cyprus	22.22	165	Mauritius	12.22
73	Maldives	22.21	166	Malawi	12.15
74	Albania	21.92	167	Haiti	12.07
75	Canada	21.85	168	Afghanistan	11.58

76	Dominican Republic	21.83	169	Niger	11.51
77	Philippines	21.81	170	Barbados	11.32
78	Cuba	21.81	171	Laos	11.23
79	Uzbekistan	21.75	172	Burundi	11.21
80	Kazakhstan	21.73	173	Gambia	11.01
81	Guatemala	21.53	174	Saint Vincent and the Grenadines	10.94
82	Togo	21.52	175	Mali	10.72
83	Bulgaria	21.46	176	Australia	10.44
84	Egypt	21.44	177	Chad	10.28
85	Morocco	21.35	178	Sierra Leone	10.02
86	Kosovo	21.26	179	Taiwan	9.98
87	Venezuela	20.76	180	New Zealand	9.80
88	Ethiopia	20.64	181	Tajikistan	8.29
89	Malaysia	20.57	182	Bhutan	6.87
90	Bolivia	20.55	183	Liberia	6.29
91	Suriname	20.54	184	Holy See	1.44
92	Botswana	20.36	185	China	0.95
93	Pakistan	20.29			

The Regional COVID-19 Severity Index is calculated based on the infection rate and growth rate of COVID-19 confirmed cases on the country level (Dong et al., 2020). The data was last updated on December 31st, 2021. Due to the large difference in data, we normalized the calculation results. The higher the index, the more severe the pandemic. The classification of countries is based on the work of Dong et al. (Dong et al., 2020). Infection rates of many overseas colonists are very high due to their too few populations, results in that other countries' indexes will be too small to distinguish. Consider that these regions do not have their own production capacity, these regions are not included in the calculation.

Table S2. Impact of commodity on EV market, Related to Figure 2.

Commodity	Impact index
Lithium mineral	100%
Cobalt ore	79%
Nickel ore	53%
Manganese ore	56%
Natural graphite	48%
Anode material	100%
Lithium carbonate	81%
Lithium hydroxide	19%
Refined cobalt	79%
Nickel chemicals	53%
EMD	56%
NCM & NCA	53%

LFP	19%
LMO	2%
LCO	25%
LIB	100%
EV	100%

EMD: electrolytic manganese dioxide; LFP: lithium iron phosphate; LCO: lithium cobalt oxide; NCM & NCA: lithium nickel cobalt manganese oxide and lithium nickel cobalt aluminum oxide; LMO: lithium manganese oxide; LIB: lithium-ion battery; EV: electric vehicle.

The impact index is quantified by proportion of EVs associated with the commodity. All EVs are powered by LIBs, thus the impact index of LIB is 100%. The market shares of various cathode materials in LIBs are: LCO, 25%; NCM & NCA, 53%; LFP, 19%; LCO, 2%. The impact index of upstream raw materials is the sum of the market shares of related downstream commodities. For example, electrolytic manganese dioxide is the raw materials that used to process NCM, NCA and LMO. Thus the impact index of electrolytic manganese dioxide is 53%+2%= 56% (the actual value contains decimals).

Table S3. Commodity Criticality Index, Related to Figure 2.

**Commodity**	**Value**
Lithium mineral	1622
Cobalt ore	1297
Nickel ore	1171
Manganese ore	1150
Natural graphite	448
Anode material	412
Lithium carbonate	1159
Lithium hydroxide	169
Refined cobalt	641
Nickel chemicals	445
EMD	500
NCM & NCA	454
LFP	23
LMO	5
LCO	181
LIB	1102
EV	1436

Commodity COVID-19 Severity Index is calculated based on the supply structure of commodities, RCSI of corresponding countries, and commodity impact on EV market. High index represents high risk of supply disruption of the commodity due to the pandemic.

Table S4. Global lithium mineral production in 2019, Related to Figure 2 and Figure 3.

**Country**	**Production/ton lithium content**
Australia	42,000

Chile	14,200
China	7,500
Argentina	6,400
Zimbabwe	1,600
USA	1,380
Portugal	1,200
Brazil	300
Canada	200
**Total**	**74,780**

Data comes from the report of United States Geological Survey (USGS) (USGS, 2020).

Table S5. Global cobalt ore production in 2019, Related to Figure 2 and Figure 3. DRC: Democratic Republic of the Congo.

Country	Production/ton cobalt content
DRC	100,000
Russia	6,100
Australia	5,100
Philippines	4,600
Cuba	3,500
Madagascar	3,300
Papua New Guinea	3,100
Canada	3,000
Zambia	3,000
Brazil	2,500
South Africa	2,400
Morocco	2,081
China	2,000
New Caledonia	1,600
Zimbabwe	888
USA	500
Finland	440
Botswana	400
Indonesia	400
Vietnam	277
**Total**	**145,186**

Data comes from the report of USGS (USGS, 2020). In this report, mine production of Morocco, Zimbabwe, Finland, Botswana, Indonesia, and Vietnam is summarized in the "Other countries" category. We divided the total output of these countries into each country according to their relative production proportion in 2015 (USGS, 2017a).

Table S6. Global nickel ore production in 2019, Related to Figure 2 and Figure 3.

Country	Production/ton

	nickel content
Indonesia	800,000
Philippines	420,000
Russia	270,000
New Caledonia	220,000
Australia	180,000
Canada	180,000
China	110,000
Brazil	67,000
South Africa	51,307
Cuba	51,045
Guatemala	47,425
Madagascar	41,700
Colombia	36,564
Papua New Guinea	25,582
Burma	24,000
USA	22,100
Greece	17,748
Botswana	15,195
Zimbabwe	14,579
Turkey	9,600
Finland	8,779
Vietnam	7,790
Serbia	7,418
Spain	7,213
Albania	5,520
Venezuela	3,700
Norway	350
Morocco	250
**Total**	**2,644,865**

Data comes from the report of USGS (USGS, 2020). In this report, mine production of South Africa
Cuba, Guatemala, Madagascar, Colombia, Papua New Guinea, Burma, USA, Greece, Botswana, Zimbabwe, Turkey, Finland, Vietnam, Serbia, Spain, Albania, Venezuela, Norway, and Morocco is summarized in the "Other countries" category. We divided the total output of these countries into each country according to their relative production proportion in 2015 (USGS, 2017b).

Table S7. Global manganese ore production in 2019, Related to Figure 2 and Figure 3.

Country	Production/ton manganese content
South Africa	5,500
Australia	3,200

Gabon	2,400
Ghana	1,400
China	1,300
Brazil	1,200
India	1,000
Ukraine	540
Malaysia	420
Cote d'Ivoire	400
Burma	210
Georgia	200
Mexico	190
Kazakhstan	130
Others	910
**Total**	**19,000**

Data comes from the report of USGS (USGS, 2020). The country "Others" includes Bulgaria, Côte d'Ivoire, Egypt, Hungary, Indonesia, Iran, Morocco, Namibia, Nigeria, Oman, Philippines, Romania, Russia (concentrate), Sudan, Thailand, Turkey, Vietnam, and Zambia. Specific output by these country was not available. In calculating the CCSI of manganese ore, the RCSI of "Others" is the average value of RCSIs of relevant countries.

Table S8. Global natural graphite production in 2019, Related to Figure 2 and Figure 3.

**Country**	**Production/ton**
China	700,000
Mozambique	100,000
Brazil	96,000
Madagascar	47,000
Canada	40,000
India	35,000
Russia	25,000
Ukraine	20,000
Norway	16,000
Pakistan	14,000
Mexico	9,000
North Korea	6,000
Vietnam	5,000
Sri Lanka	4,000
Namibia	2,220
Turkey	2,000
Zimbabwe	2,000
Austria	1,000
Germany	180
Uzbekistan	20
**Total**	**1,124,420**

Data comes from the report of USGS (USGS, 2020).

Table S9. Global anode material production in 2019, Related to Figure 2 and Figure 3.

Country	Production/ton
China	265,000
Japan	41,400
Korea, South	19,600
**Total**	**326,000**

Data of China's production and global total production come from the report of Huajing Industrial Research Institute (Huajing Industrial Research Institute, 2020b). Japan's production is assumed to be the total production of Hitachi Chemical and Mitsubishi Chemical, ignoring that of other small firms. South Korea's production is the global total production minus China's and Japan's.

Table S10. Global lithium carbonate production in 2019, Related to Figure 2 and Figure 3.

Country	Production/ton
China	154,000
Chile	102,692
Argentina	34,904
**Total**	**291,596**

Data of China's production comes from the report of China Bulk Commodity (CBC, 2021). Data of Chile's and Argentina's production come from the declared production in the enterprise public reports of SQM, Albemarle, Orocobre, and Livent (FMC).

Table S11. Global lithium hydroxide production in 2019, Related to Figure 2 and Figure 3.

Country	Production/ton
China	76,000
USA	13,173
Chile	9,120
Russia	3,040
**Total**	**101,333**

Data comes from the report of China Industrial Information Network (CHYXX, 2020a).

Table S12. Global refined cobalt production in 2019, Related to Figure 2 and Figure 3.

Country	Production/ton
China	78,360
Finland	12,800
Belgium	6,600
Canada	6,500
Japan	4,000
Norway	3,500

Australia	3,200
Madagascar	2,800
Zambia	2,000
Russia	1,800
Morocco	1,600
South Africa	1,000
DRC	400
India	100
**Total**	**124,660**

Data of global total production comes from China Industrial Information Network (CHYXX, 2020c). Production on the country level is estimated based on the production share of each country in 2016 and global total production in 2019, assuming that supply structure was unchanged. The production share of each country in 2016 comes from the report of Cobalt Institute (Cobalt Institute, 2020).

Table S13. Global nickel chemical production in 2019, Related to Figure 2 and Figure 3.

**Country**	**Production/kiloton**
China	13.05
Europe	2.8
Taiwan	1.8
Japan	1.65
Korea, South	1.2
Others	0.6
**Total**	**21.1**

Data comes from the report of Wood Mackenzie (Wood Mackenzie, 2020).

Table S14. Global EMD production in 2019, Related to Figure 2 and Figure 3.

**Country**	**Production/ton**
China	300,400
USA	50,143
Japan	28,286
Greece	22,286
Spain	12,857
Colombia	5,143
India	857
**Total**	**419,971**

Data comes from the report of Huajing Industrial Research Institute (Huajing Industrial Research Institute, 2020a).

Table S15. Global NCM & NCA production in 2019, Related to Figure 2 and Figure 3.

**Country**	**Production/ton**
China	197,000
Korea, South	94,135

Japan	56,481
**Total**	**347,615**

Data of China's production comes from the report of Xinluo Information (Shanghai Xinluo Network Technology, 2020b). Data of global total production comes from the report of Gaogong Industrial Research Consulting (GGII, 2020). The production of South Korea and Japan is estimated based on their relative production proportion of NCM & NCA in 2017 (Sun et al., 2019).

Table S16. Global LFP production in 2019, Related to Figure 2 and Figure 3.

Country	Production/ton
China	80,000
Canada	5,000
USA	4,000
**Total**	**89,000**

Data of China's production comes from the report of Xinluo Information (Shanghai Xinluo Network Technology, 2020c). Canada's and USA's production is the data for the year of 2015 (Sun et al., 2018). In recent years, there has been no expansion of LFP production capacity in other countries except China.

Table S17. Global LMO production in 2019, Related to Figure 2 and Figure 3.

Country	Production/ton
China	76,400
Japan	10,629
**Total**	**87,029**

Data of China's production comes from the report of Xinluo Information (Shanghai Xinluo Network Technology, 2020a). Japan's production is the data for the year of 2017 (Sun et al., 2018). In recent years, there has been no expansion of LMO production capacity in other countries except China.

Table S18. Global LCO production in 2019, Related to Figure 2 and Figure 3.

Country	Production/ton
China	54,800
Korea, South	23,413
Japan	5,509
**Total**	**83,721**

Data comes from the report of China Industrial Information Network (CHYXX, 2020b).

Table S19. Global LIB production in 2019, Related to Figure 2 and Figure 3.

Country	Production/GWh
China	132
USA	35
Korea, South	13
Japan	13

Hungary	8
Poland	6
United Kingdom	3
**Total**	**208**

Data of China's production comes from the report of National Bureau of Statistics of China (NBSC, 2020). Data of others' production comes from the report of Juda Large (Juda LARGE, 2020).

Table S20. Global EV production in 2019, Related to Figure 2 and Figure 3.

**Country**	**Production/unit**
China	1,242
USA	544
Germany	202
Japan	143
France	116
Korea, South	111
Slovakia	21
Austria	13
Spain	11
Belgium	6
Slovenia	4
Sweden	2
**Total**	**2,358**

The EV production on the country level is estimated based on the international trade data and sales data:

$$\text{Production} = \text{Sales} + \text{Export} - \text{Import}.$$

EV sales on the country level comes from the report of International Energy Agency (IEA, 2020). The international trade data comes from the UNComtrade database, using the custom code of 870360, 870370, and 870380 (UN Comtrade, 2020).

Table S21. The timing of the regional lockdown measures taken in 2020, Related to Figure 4.

**Region**	**Country**	**Lockdown starting time**	**Lockdown end time**	**Duration**
Europe	Austria	2020/3/15	2020/4/14	30
Europe	Belgium	2020/3/18	2020/5/18	61
Europe	Bulgaria	2020/3/13	2020/5/13	61
Europe	Czech Republic	2020/3/12	2020/5/11	60
Europe	Denmark	2020/3/12	2020/4/15	34
Europe	Estonia	2020/3/13	2020/5/17	65
Europe	Finland	2020/3/16	2020/5/31	76
Europe	France	2020/3/17	2020/5/11	55
Europe	Germany	2020/3/18	2020/5/4	47

Europe	Hungary	2020/3/11	2020/6/18	99
Europe	Italy	2020/3/10	2020/5/4	55
Europe	Latvia	2020/3/15	2020/6/10	87
Europe	Lithuania	2020/3/16	2020/6/1	77
Europe	Luxembourg	2020/3/17	2020/5/7	51
Europe	Moldova	2020/3/16	2020/5/31	76
Europe	North Macedonia	2020/3/18	2020/6/13	87
Europe	Poland	2020/3/13	2020/4/30	48
Europe	Portugal	2020/3/13	2020/5/2	50
Europe	Romania	2020/3/16	2020/5/14	59
Europe	Russia	2020/3/18	2020/5/11	54
Europe	Serbia	2020/3/15	2020/5/6	52
Europe	Slovakia	2020/3/15	2020/5/11	57
Europe	Spain	2020/3/14	2020/5/4	51
Europe	Switzerland	2020/3/16	2020/6/19	95
Europe	UK	2020/3/23	2020/6/21	90
Europe	Ukraine	2020/3/16	2020/5/29	74
Asia	Armenia	2020/3/16	2020/6/13	89
Asia	China	2020/1/25	2020/5/2	98
Asia	Cyprus	2020/3/15	2020/6/9	86
Asia	Israel	2020/3/18	2020/5/3	46
Asia	India	2020/3/24	2020/5/11	48
Asia	Japan	2020/4/7	2020/5/25	48
Asia	Jordan	2020/3/17	2020/5/3	47
Asia	Kazakhstan	2020/3/16	2020/5/11	56
Asia	Korea	2020/3/3	2020/5/6	64
Asia	Kuwait	2020/3/11	2020/5/31	81
Asia	Kyrgyzstan	2020/3/17	2020/5/1	45
Asia	Lebanon	2020/3/15	2020/5/4	50
Asia	Malaysia	2020/3/18	2020/5/8	51
Asia	Palestine	2020/3/6	2020/5/4	59
Asia	Philippines	2020/3/10	2020/6/1	83
Asia	Singapore	2020/3/23	2020/6/2	71
Asia	Vietnam	2020/3/22	2020/5/1	40
Oceania	Australia	2020/3/20	2020/5/8	49
Oceania	New Zealand	2020/3/19	2020/5/11	53
America	Argentina	2020/3/12	2020/5/10	59
America	Brazil	2020/3/30	2020/6/1	63
America	Canada	2020/3/16	2020/5/4	49
America	Chile	2020/3/19	2020/5/1	43
America	Columbia	2020/3/17	2020/5/11	55
America	Costa Rica	2020/3/16	2020/5/11	56
America	Cuba	2020/3/24	2020/6/11	79

America	Dominica	2020/3/19	2020/6/1	74
America	Ecuador	2020/3/16	2020/5/11	56
America	Guatemala	2020/3/5	2020/6/1	88
America	Honduras	2020/3/17	2020/5/14	58
America	Panama	2020/3/13	2020/6/1	80
America	Peru	2020/3/15	2020/5/24	70
America	Salvador	2020/3/14	2020/6/16	94
America	USA	2020/3/11	2020/5/1	51
America	Venezuela	2020/3/16	2020/6/14	90
Africa	Cote d'Ivoire	2020/3/22	2020/5/15	54
Africa	Djibouti	2020/3/15	2020/5/16	62
Africa	Egypt	2020/3/19	2020/6/4	77
Africa	Libya	2020/3/14	2020/6/15	93
Africa	Namibia	2020/3/17	2020/6/23	98
Africa	South Africa	2020/3/15	2020/5/1	47
Africa	Sudan	2020/3/16	2020/5/6	51
Africa	Tunisia	2020/3/20	2020/5/4	45
Africa	Zimbabwe	2020/3/17	2020/5/17	61
**Average**				**64**

The information is collected from the public announcements by governments around the world. In some countries, the lockdown measures are implemented in a hierarchical and phased manner. In addition, different administrative regions within the same country may take lockdown measures at different times. Here, we set the beginning of each country' lockdown measure as the first time that a local lockdown is announced within a country, and the end time as the latest time for a local government to announce the lifting of the lockdown.

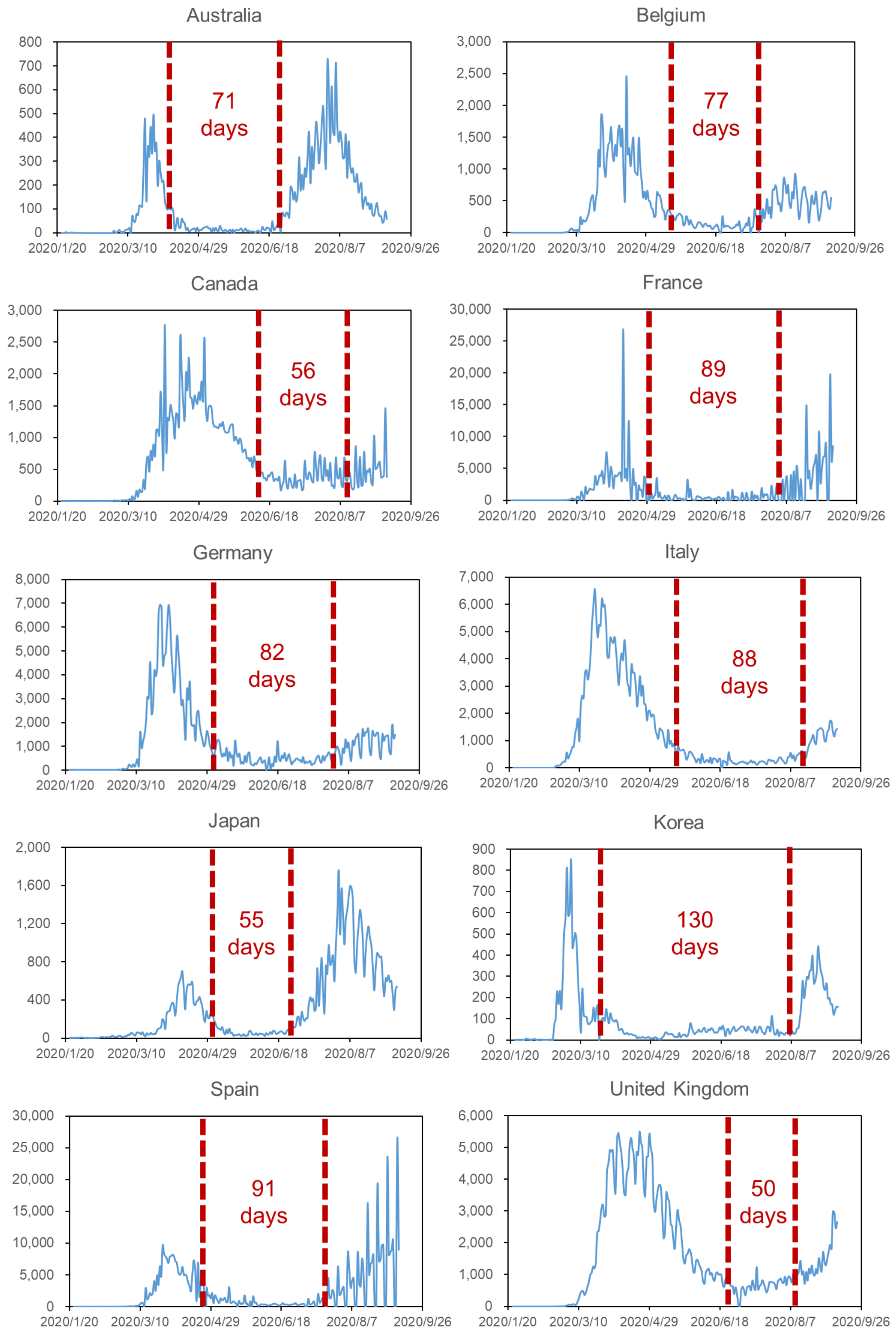

Figure S1. Time interval between the first and the second outbreak of COVID-19 in typical countries, Related to Figure 4.

Blue lines show the newly confirmed cases. Red dotted lines show the end time of the first outbreak (newly confirmed cases dropped below 10% of its first peak) and the beginning time of the second outbreak (newly confirmed cases reached 10% of its second peak). Typical countries refer to the countries that have certain nucleic acid testing capabilities and have

contained the first wave of pandemic and are suffering the second wave of pandemic. For example, the USA is not selected as a typical country because the number of people diagnosed in the country has never fallen below 10% of its first peak even though it has adequate virus detection capacity.

Table S22. Production capacity planning of LIB companies, Related to Figure 3.

Country affiliation of company	Production location	Production capacity 2019/GWh	Scheduled production capacity/GWh	Final release time	Expected additional production capacity in 2030/GWh
CATL	China	58	103	2023	520
	China		52	2023	
	Germany	14	64	2025	100
Panasonic	China	5	12		
	China		30		
	USA	30	54		
LG Chem	Korea	4	4		110
	USA	2.4	2.4		
	Poland	30	65	2023	
	China	3	32	2023	
BYD	China	24	24		
	China	2	2		
	China	14	14		
	China		20		
	China		30		
	China		20		
Envision AESC	Japan & USA & UK	7.5	7.5		
	China		20		
Gotion High-Tech	China	3	3		300
	China	2	2		
	China	3	3		
	China	2	15		
	China		10		
PEVE	China		25		
Tianjin Lishen	China	10	30	2025	100
SKI	Korea	6	6		100
	Hungary	7.5	23.5		
	China	7.5	7.5		
	USA		10	2022	

Blackstone	Germany	2		
Indonesia government	Indonesia	27		
MARii	Malaysia	5		
Northvolt	Sweden	32	2023	
Verkor	France	50		
Automotive Cells Company	France & Germany	24	2023	48
Blackstone	Germany	2		
Tesla	USA	10	2022	100
SVOLT	China			600
AVIC Innovation Capital	China			500

The production capacity planning information is collected from the public statements issued by the companies concerned. The data was last updated on December 31th, 2020.

Table S23. Production capacity planning of NCM & NCA companies, Related to Figure 3.

Country affiliation of company	Company	Production location	Scheduled production/ton
Belgium	Umicore	Poland	50,000
Germany	BASF	Poland	40,000
UK	Johnson Matthey	Poland	100,000
China	CATL	China	100,000
China	Ningbo Shanshan	China	28,800
China	Xiamen Tungsten	China	20,000
China	Langsheng Technology	China	20,000
China	Huayou Cobalt	China	100,000
China	LG Chem	Korea	120,000

The production capacity planning information is collected from the public statements issued by the companies concerned. The data was last updated on December 31th, 2020.

Table S24. Production capacity planning of lithium carbonate companies, Related to Figure 3.

Country affiliation of company	Company	Production location	Production 2019/ton	Scheduled production before 2030/ton
China	Tianqi Lithium	China	27,500	100,000
China	Qinghai HXR Lithium Tech	China	20,000	20,000
China	Ganfeng Lithium	China	36,000	36,000

China	Sichuan Yahua Industrial Group	China	10,300	10,300
China	Qinghai Salt Lake Industry Group	China	30,000	60,000
China	Qinghai Lithium	China	10,000	27,000
China	Qinghai Dongtai Jinier Lithium Resources	China	20,000	20,000
China	Jiangxi Hezong Lithium Tech	China	8,000	8,000
China	Bioway Biotechnology	China	8,000	8,000
China	Shandong Ruifu Lithium	China	8,000	8,000
China	Jiangsu Ronghui General Lithium	China	6,000	6,000
China	Sichuan Nike Guorun Tech	China	5,000	5,000
China	Xizang Guoneng Mineral	China	5,000	5,000
China	Yichun Yinli New Energy	China	5,000	5,000
China	CITIC Guoan Group	China	5,000	15,000
China	Dahua Chem	China	5,000	15,000
China	Jiangxi Albemarle	China	3,600	3,600
China	Rongjie	China	3,000	3,000
China	Xizang Urban Development Investment	China	5,000	45,000
China	Xizang Mineral	China	8,000	8,000
China	Minmetals Salt Lake	China	2,400	32,400
China	Qinghai Bohua	China	2,000	2,000
China	Zangge Holdings	China	20,000	20,000
China	Qinghai Jintai Lithium	China	10,000	10,000
Chile	SQM	Chile	148,000	198,000
USA	ALB	Chile	30,000	70,000
Australia	Orocobre	Argentina	17,500	42,500
USA	Livent（FMC）	Argentina	43,000	52,500
Australia	Galaxy Resources	Argentina		25,000
**Total**			**501,300**	**860,300**

The production capacity planning information is collected from the public statements issued by the companies concerned. The data was last updated on December 31th, 2020.

Table S25. Production capacity planning of lithium hydroxide companies, Related to Figure 3.

Country affiliation of company	Company	Production location	Scheduled production before 2030/ton
USA	ALB & Mineral resource	Australia-Kemerton	100,000
USA	Livent	USA-Bessermer	5,000
Australia	Orocobre	Japan-Naraha	10,000

The production capacity planning information is collected from the public statements issued by the companies concerned. The data was last updated on December 31th, 2020.

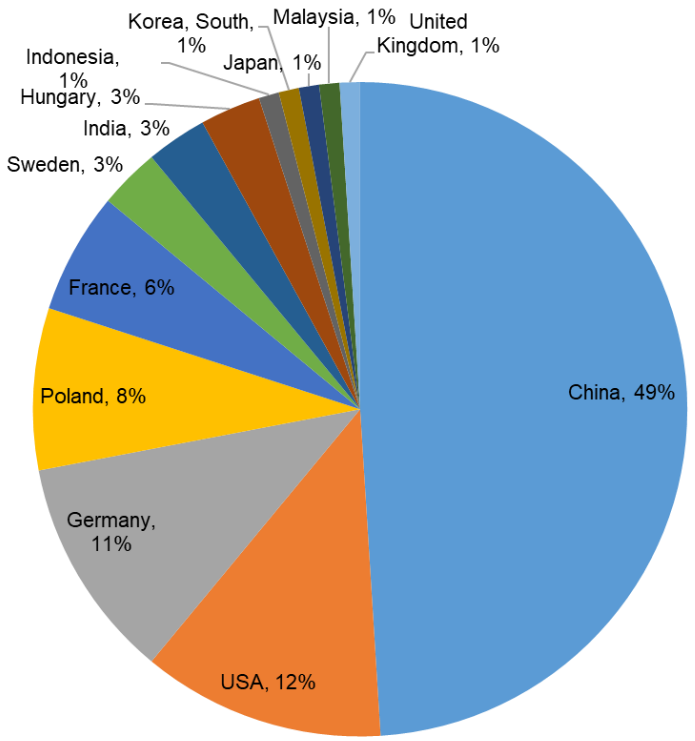

Figure S2. Estimated global production structure of LIB in 2030, Related to Figure 3.
Global production structure of LIB in 2030 is estimated based on the production capacity planning of LIB companies shown in the Table S20.

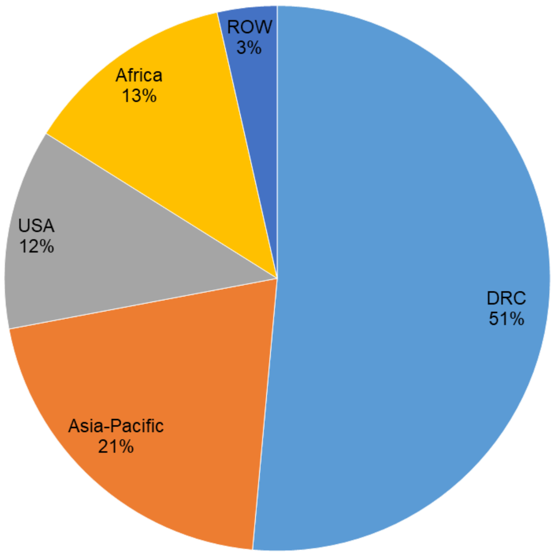

Figure S3. Estimated global production structure of cobalt ore in 2030, Related to Figure 3.

The estimation of global production structure of cobalt ore in 2030 is adopted from the results in the study of Fu et al. (Fu et al., 2020).

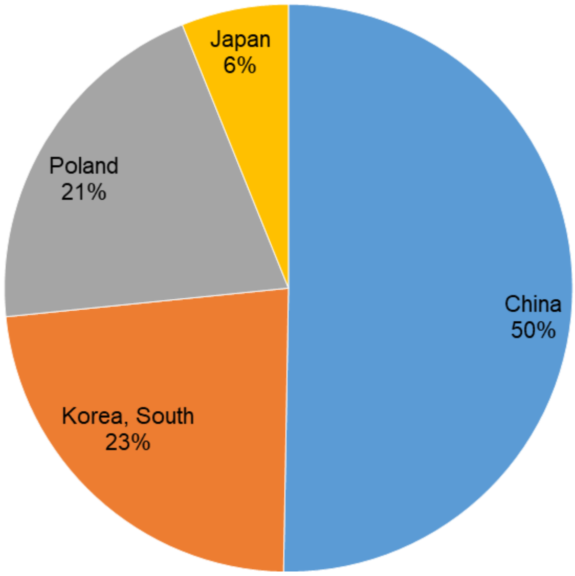

Figure S4. Estimated global production structure of NCM & NCA in 2030, Related to Figure 3.

Global production structure of NCM & NCA in 2030 is estimated based on the production capacity planning of NCM & NCA companies shown in the Table S21.

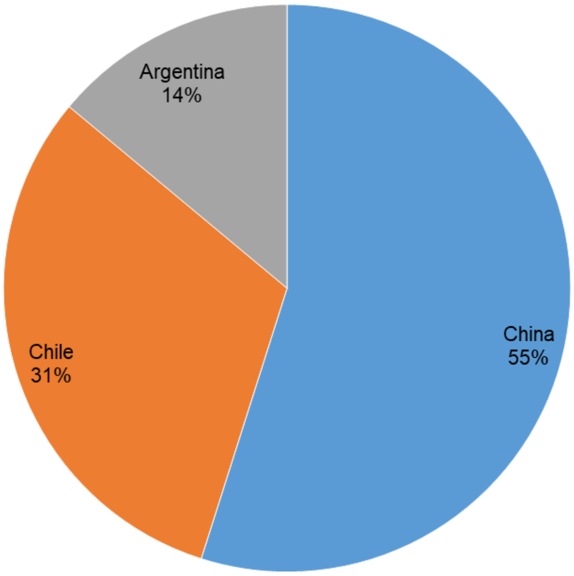

Figure S5. Estimated global production structure of lithium carbonate in 2030, Related to Figure 3.

Global production structure of lithium carbonate in 2030 is estimated based on the production capacity planning of lithium carbonate companies shown in the Table S22.

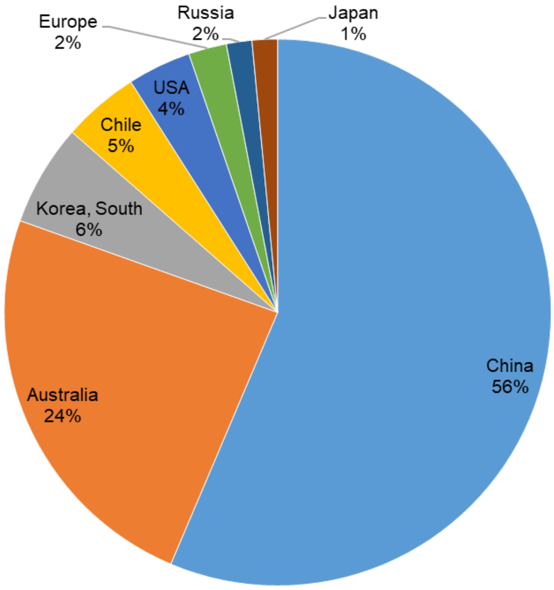

Figure S6. Estimated global production structure of lithium hydroxide in 2030, Related to Figure 3.

Global production structure of lithium hydroxide in 2030 is estimated based on the production capacity planning of lithium hydroxide companies shown in the Table S23.

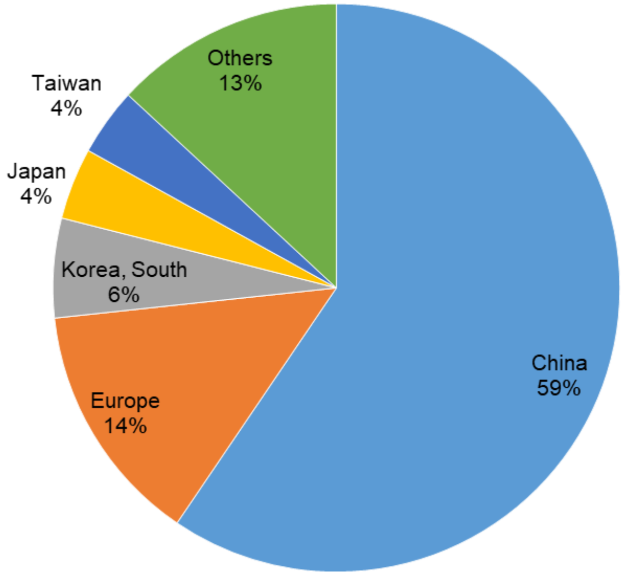

Figure S7. Estimated global production structure of nickel chemicals in 2030, Related to Figure 3.

The estimation of global production structure of nickel chemicals in 2030 is adopted from the report of Wood Mackenzie (Wood Mackenzie, 2020).

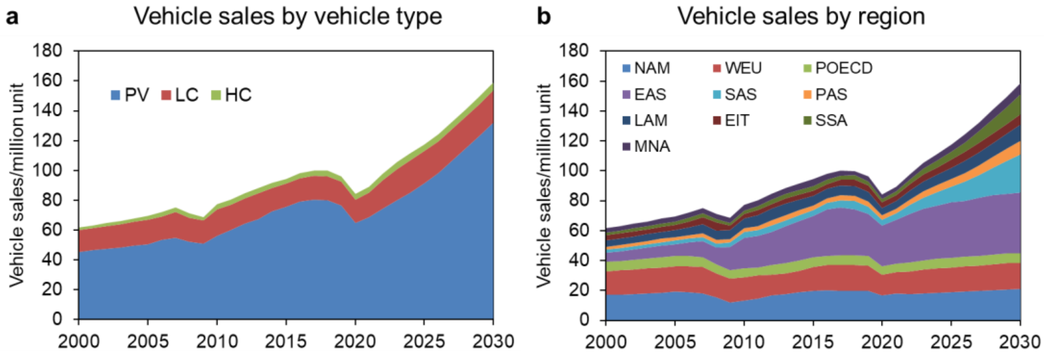

Figure S8. Vehicle sales by vehicle type and region, Related to Figure 5. PV: passenger vehicle; LC: light-duty commercial vehicle; HC: heavy-duty commercial vehicle; NAM: North America; WEU: Western Europe; POECD: Pacific countries of Organization for Economic Co-operation and Development; EAS: East Asia; SAS: South Asia; PAS: South-East Asia and Pacific; LAM: Latin America and Caribbean; EIT: Economies in Transition; SSA: Sub-Saharan Africa; MNA: Middle East and North Africa.

Subfigures indicate vehicle sales by vehicle type (**a**) and by region (**b**). Vehicle sales is modeled for all countries separately considering the differences between vehicle types (PV, LC, and HC). To simplify the chart, the countries are grouped into 10 regions. The region categorization is based on IPCC RC10 (IPCC, 2015). The total vehicles sales is the same for three scenarios.

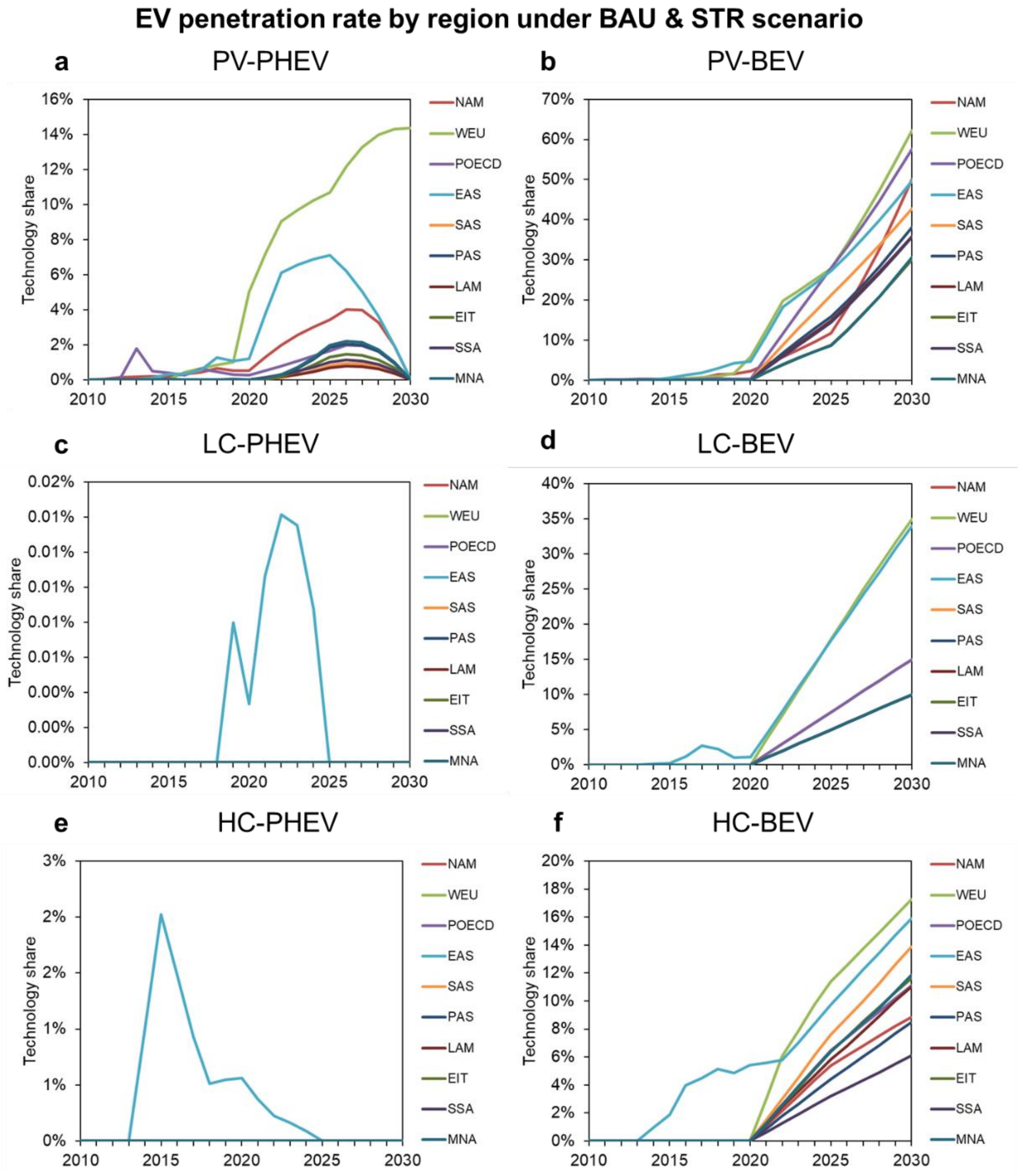

Figure S9. EV sales penetration rate by region under BAU & STR scenario, Related to Figure 5. BAU: business-as-usual; STR: short-term recovery. BEV: battery electric vehicle; PHEV: plug-in hybrid electric vehicle.

Subfigures indicate penetration rate of plug-in hybrid electric passenger vehicle (**a**), battery electric passenger vehicle (**b**), plug-in hybrid electric light-duty commercial vehicle (**c**), plug-in hybrid electric light-duty commercial vehicle (**d**), plug-in hybrid electric heavy-duty commercial vehicle (**e**), and battery electric heavy-duty commercial vehicle (**f**). The EV sales penetration rate is modeled for all countries separately considering the differences among vehicle types. The EV sales penetration rate is the same for BAU and STR scenario. Although the production capacity of LIBs in the STR scenario is reduced, the reduction is not significant enough to constrain the growth of demand for EVs.

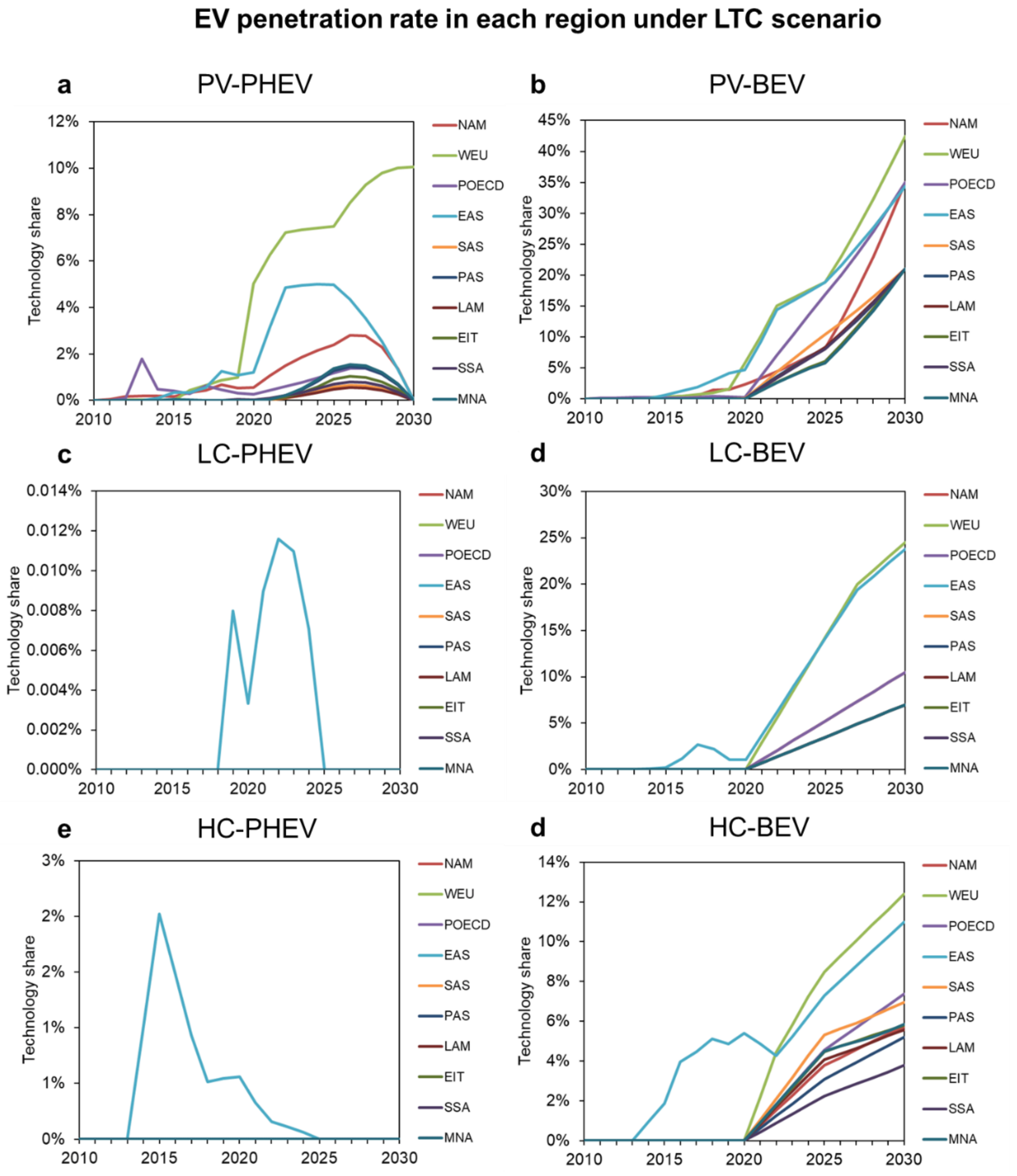

Figure S10. EV sales penetration rate by region under LTC scenario, Related to Figure 5.
LTC: long-term coexistence.
Subfigures indicate penetration rate of plug-in hybrid electric passenger vehicle (**a**), battery electric passenger vehicle (**b**), plug-in hybrid electric light-duty commercial vehicle (**c**), plug-in hybrid electric light-duty commercial vehicle (**d**), plug-in hybrid electric heavy-duty commercial vehicle (**e**), and battery electric heavy-duty commercial vehicle (**f**).

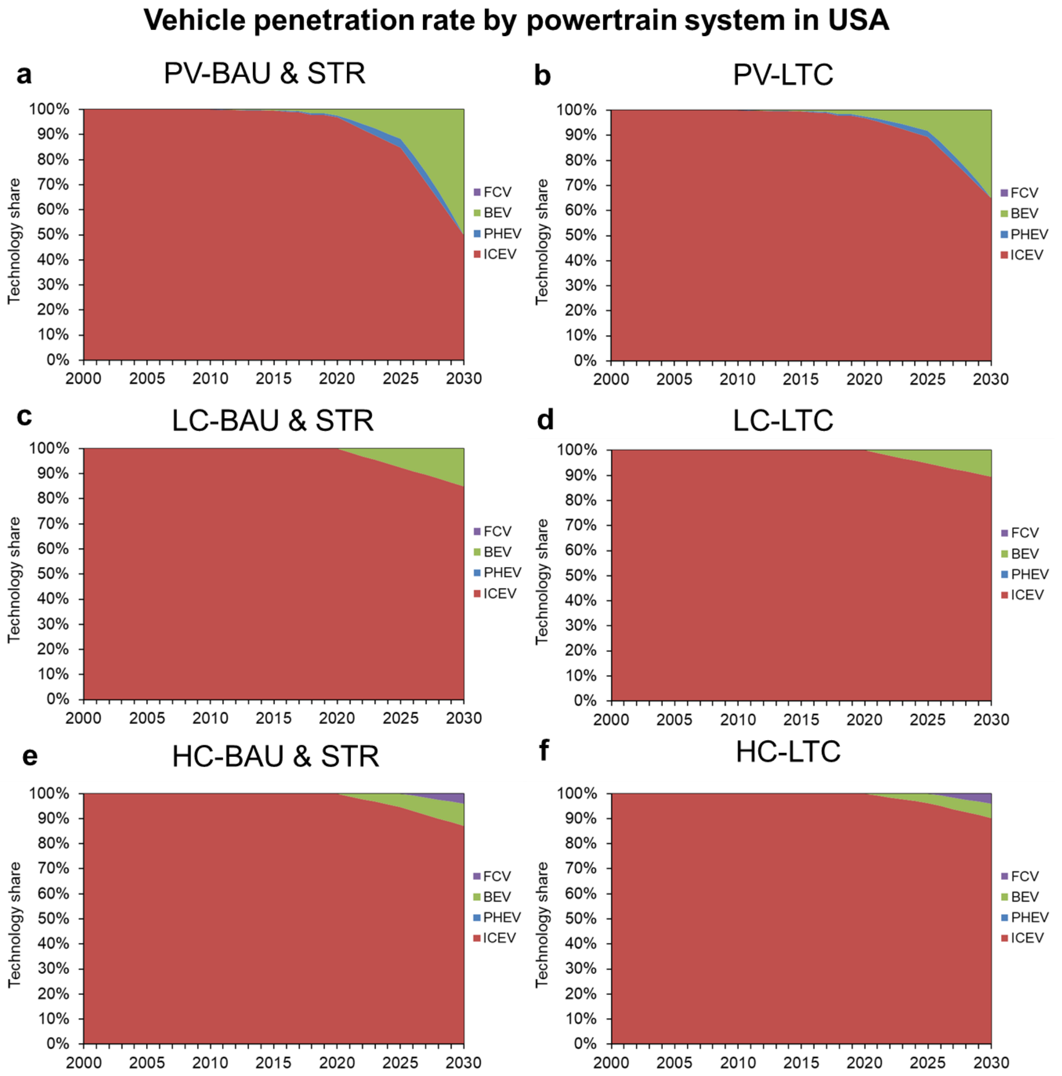

Figure S11. Vehicle penetration by powertrain system in USA, Related to Figure 5. ICEV: internal combustion engine vehicle (including hybrid electric vehicle); FCV: fuel cell vehicle. Subfigures indicate vehicle sales penetration in the USA for passenger vehicle under the BAU & STR scenarios (**a**), passenger vehicle under the LTC scenario (**b**), light-duty commercial vehicle under the BAU & STR scenarios (**c**), light-duty commercial vehicle under the LTC scenario (**d**), heavy-duty commercial vehicle under the BAU & STR scenarios (**e**), heavy-duty commercial vehicle under the LTC scenario (**f**). The vehicle penetration in four powertrain system types is modeled for all countries separately considering the differences between vehicle types. Here the USA is chosen as the typical representative of developed countries to show.

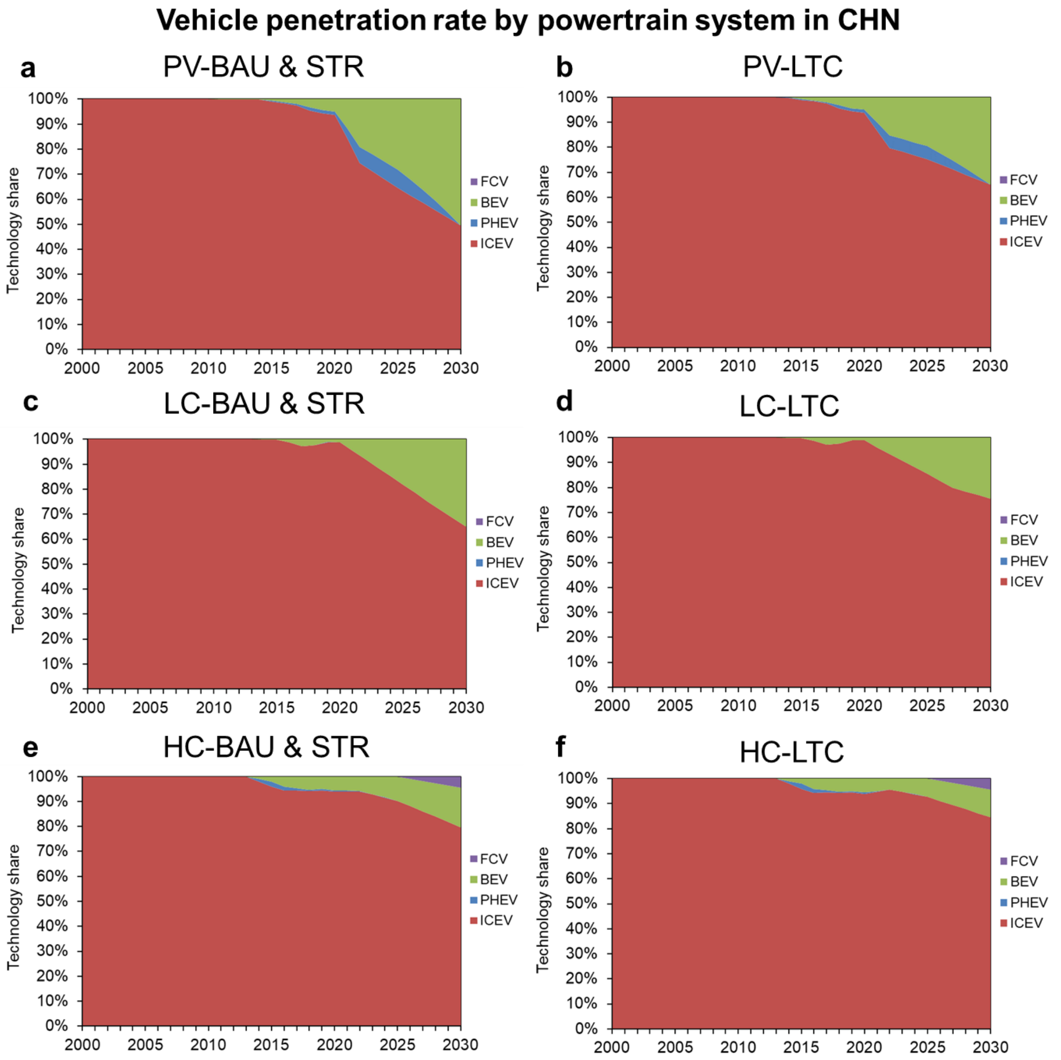

Figure S12. Vehicle penetration of LDVs in China, Related to Figure 5. ICEV: internal combustion engine vehicle; HEV: hybrid electric vehicle; FCV: fuel cell vehicle.

Subfigures indicate vehicle sales penetration in China for passenger vehicle under the BAU & STR scenarios (**a**), passenger vehicle under the LTC scenario (**b**), light-duty commercial vehicle under the BAU & STR scenarios (**c**), light-duty commercial vehicle under the LTC scenario (**d**), heavy-duty commercial vehicle under the BAU & STR scenarios (**e**), heavy-duty commercial vehicle under the LTC scenario (**f**). The vehicle penetration in four powertrain system types is modeled for all countries separately considering the differences between vehicle types. Here China is chosen as the typical representative of developing countries to show.

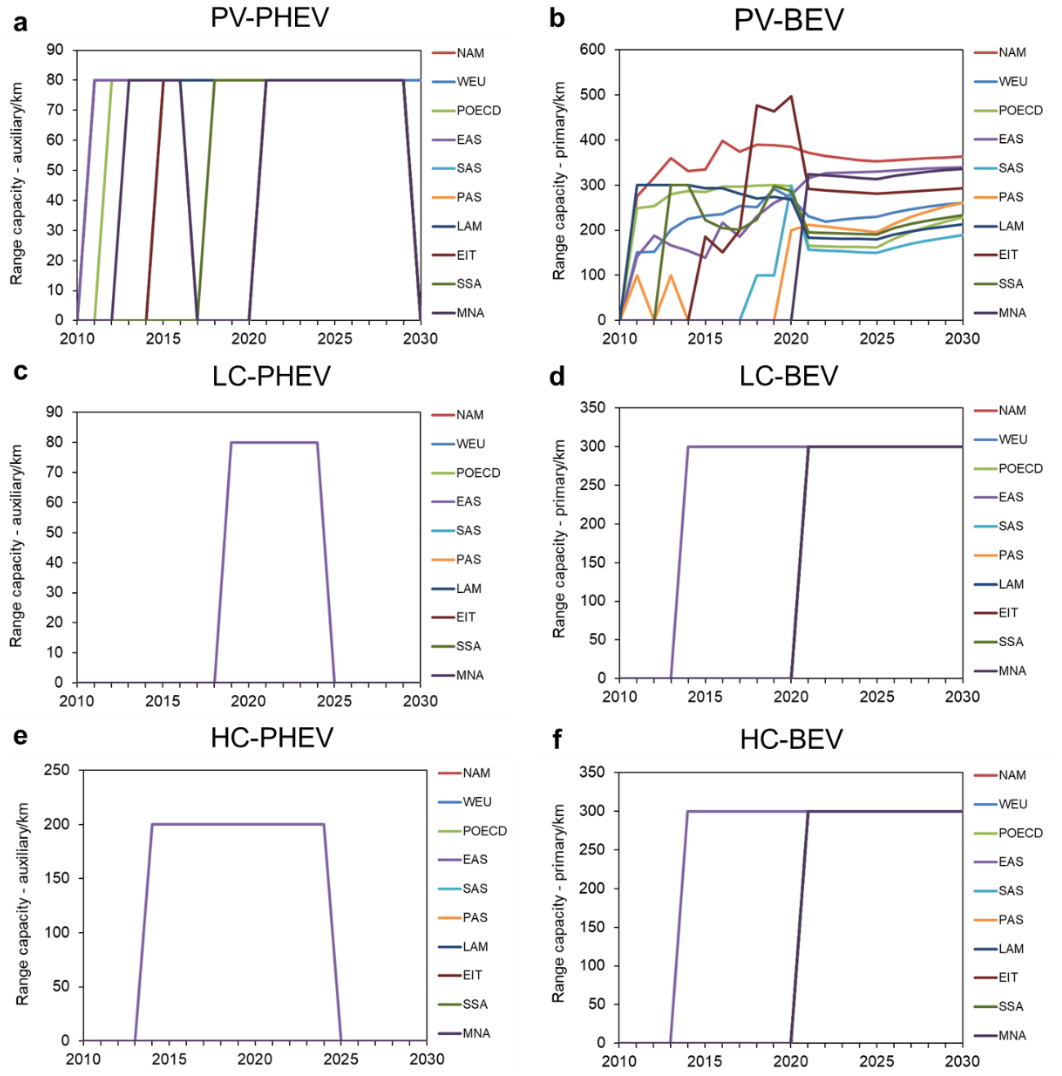

Figure S13. The vehicle electric range, Related to Figure 5.

Subfigures indicate vehicle electric range of plug-in hybrid electric passenger vehicle (**a**), battery electric passenger vehicle (**b**), plug-in hybrid electric light-duty commercial vehicle (**c**), plug-in hybrid electric light-duty commercial vehicle (**d**), plug-in hybrid electric heavy-duty commercial vehicle (**e**), and battery electric heavy-duty commercial vehicle (**f**). This factor is the same for three scenarios. The electric range for PHEVs is the range when vehicles are driven only by the electric motors.

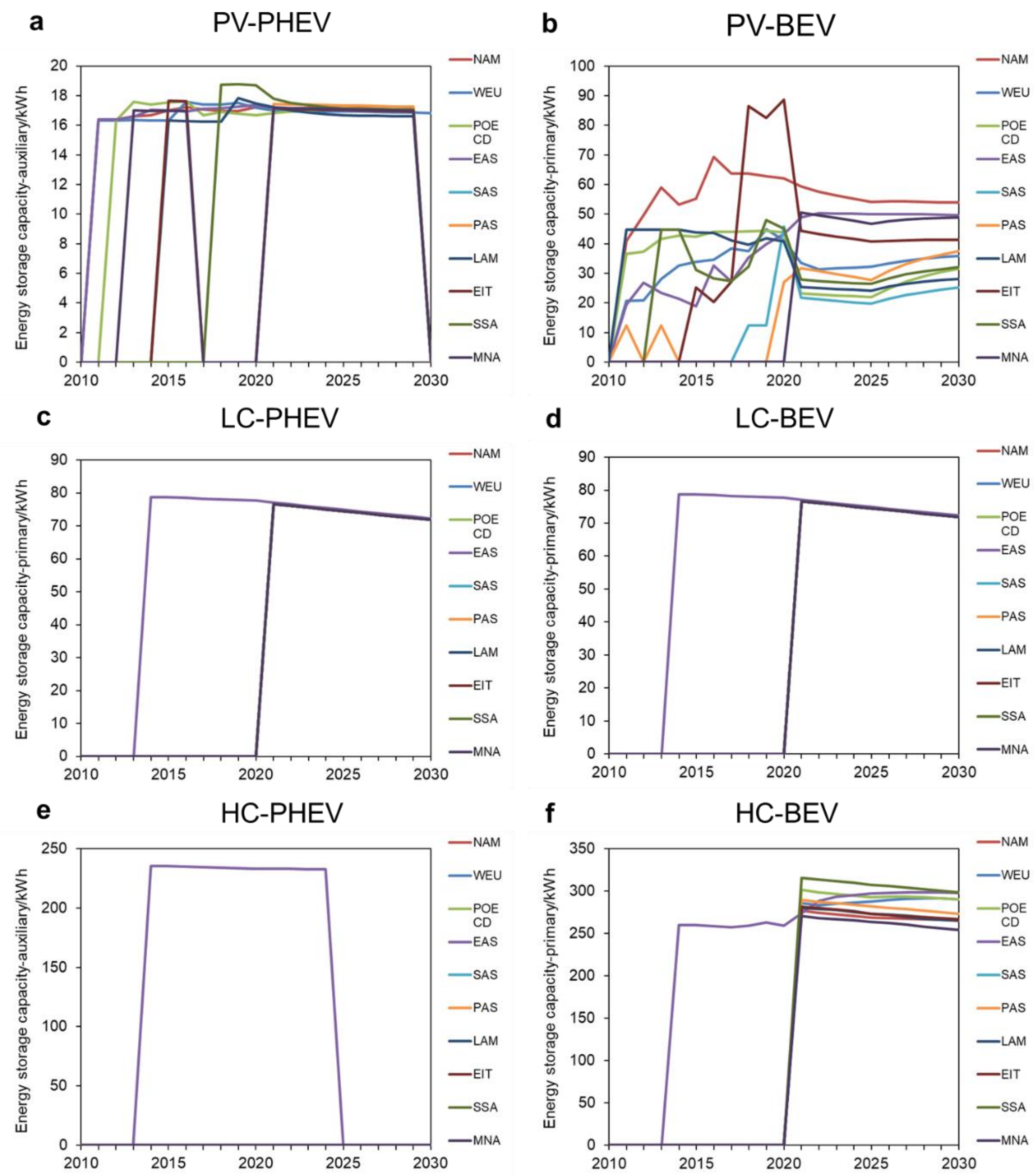

Figure S14. The battery load of BEVs and PEHVs, Related to Figure 5.

Subfigures indicate battery load of plug-in hybrid electric passenger vehicle (**a**), battery electric passenger vehicle (**b**), plug-in hybrid electric light-duty commercial vehicle (**c**), plug-in hybrid electric light-duty commercial vehicle (**d**), plug-in hybrid electric heavy-duty commercial vehicle (**e**), and battery electric heavy-duty commercial vehicle (**f**). This factor is the same for three scenarios.

EV sales under BAU & STR scenario

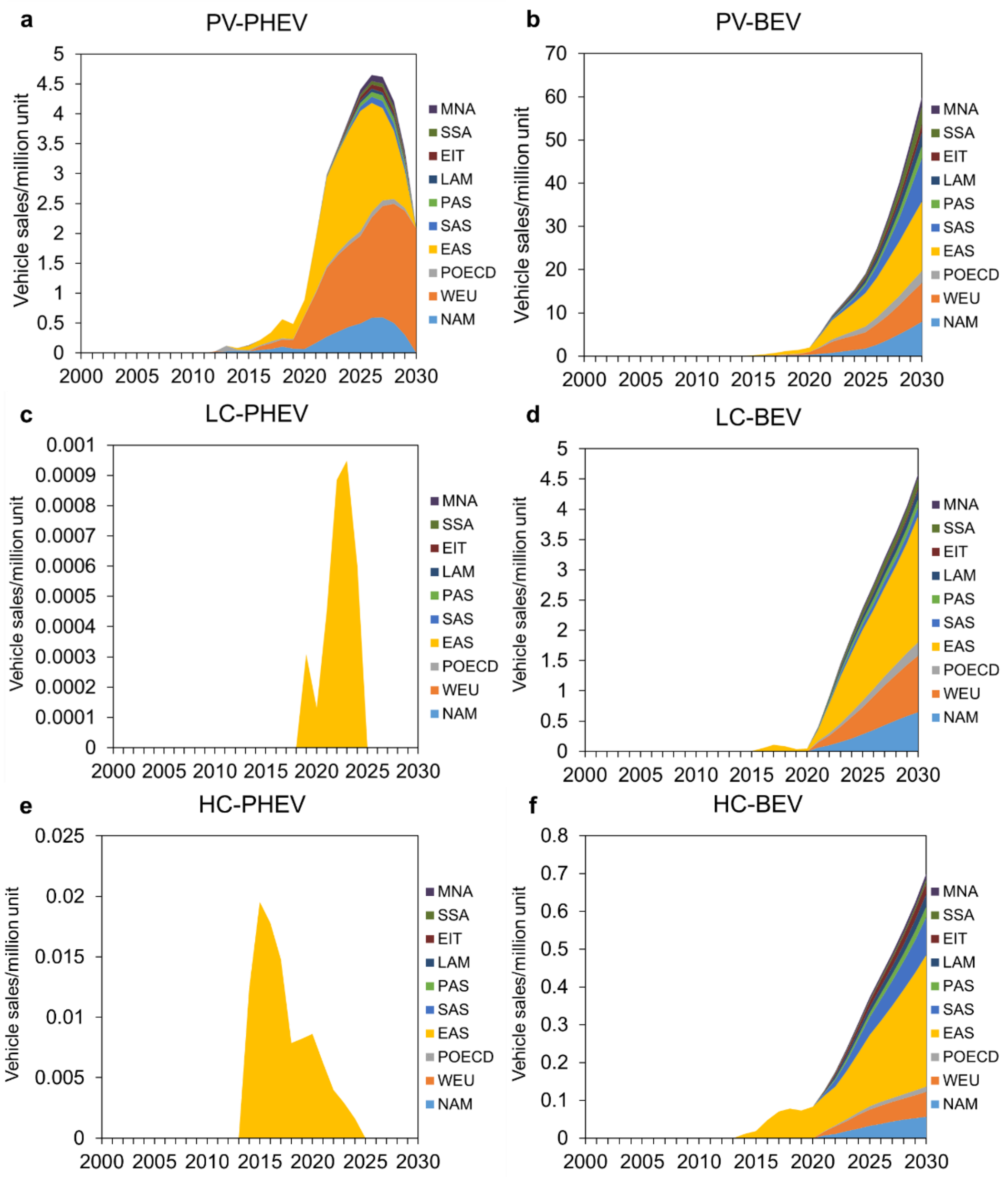

Figure S15. BEV and PHEV sales under BAU & STR scenario, Related to Figure 5. Subfigures indicate sales under BAU & STR scenarios of plug-in hybrid electric passenger vehicle (**a**), battery electric passenger vehicle (**b**), plug-in hybrid electric light-duty commercial vehicle (**c**), plug-in hybrid electric light-duty commercial vehicle (**d**), plug-in hybrid electric heavy-duty commercial vehicle (**e**), and battery electric heavy-duty commercial vehicle (**f**).

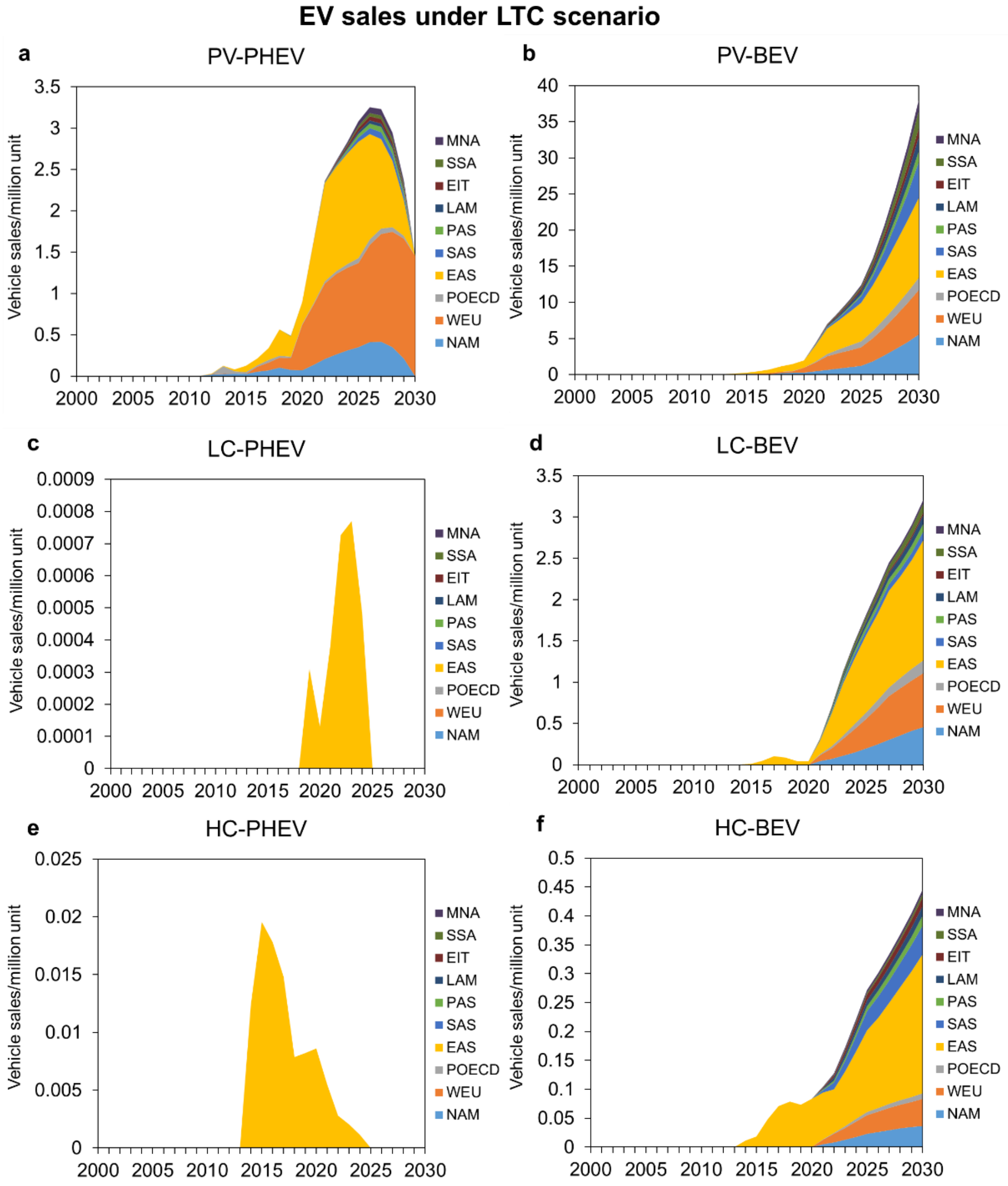

Figure S16. BEV and PHEV sales under LTC scenario, Related to Figure 5.

Subfigures indicate sales under LTC scenario of plug-in hybrid electric passenger vehicle (**a**), battery electric passenger vehicle (**b**), plug-in hybrid electric light-duty commercial vehicle (**c**), plug-in hybrid electric light-duty commercial vehicle (**d**), plug-in hybrid electric heavy-duty commercial vehicle (**e**), and battery electric heavy-duty commercial vehicle (**f**).

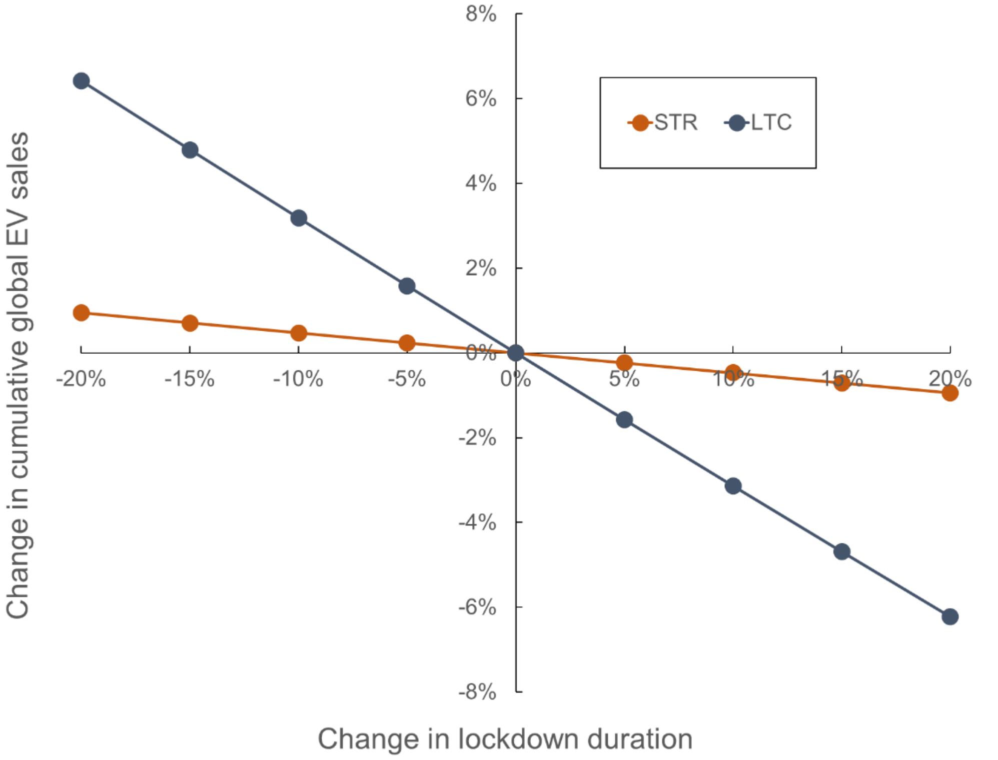

Figure S17. Uncertainty analysis of lockdown period, Related to Table 1 and Figure 5. Figure shows change in global cumulative EV sales from 2020 to 2030 with change of "lockdown duration" parameter under the STR and LTC scenarios, respectively.

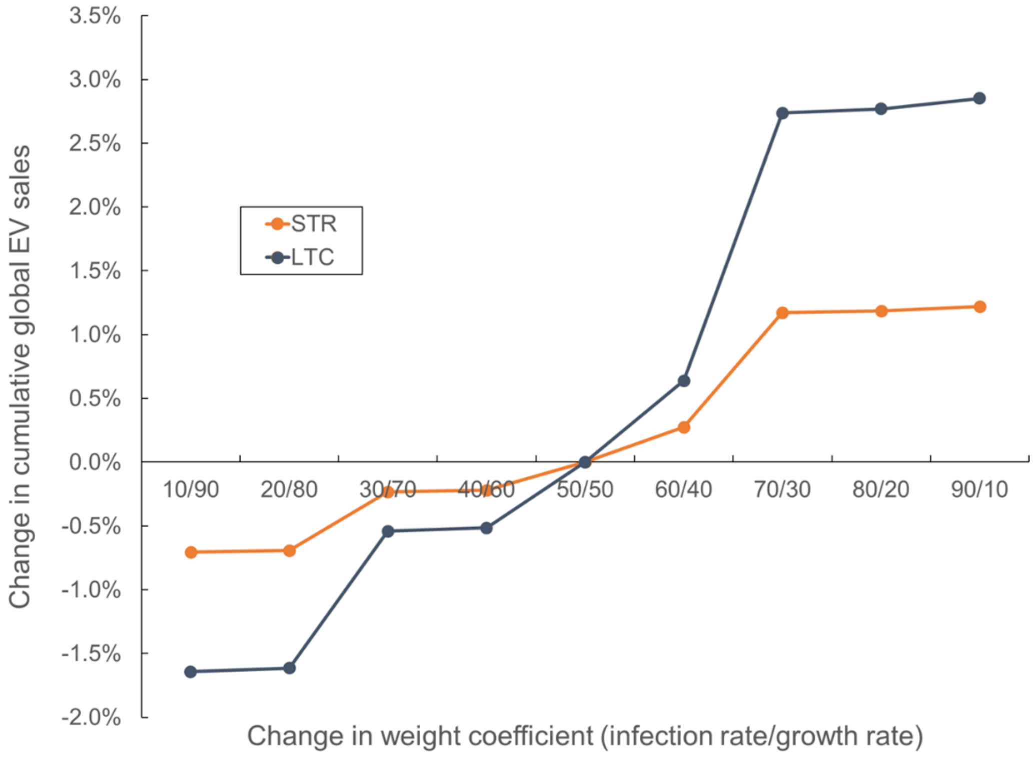

Figure S18. Uncertainty analysis of weight coefficients of infection rate and growth rate in RCSI, Related to Figure 2.

Figure shows change in global cumulative EV sales from 2020 to 2030 with change of “weight coefficients of infection rate and growth rate in RCSI” under the STR and LTC scenario, respectively.

References

CBC, 2021. Lithium industry statistics. China Bulk Commodity, http://www.cbcie.com.

CHYXX, 2020a. Analysis of global and Chinese lithium hydroxide industry sales volume, export situation and capacity distribution in 2019. China Industrial Information Network, https://www.chyxx.com/industry/202001/829070.html.

CHYXX, 2020b. China and overseas new energy vehicle battery demand and cathode material development status analysis. China Industrial Information Network, https://www.chyxx.com/industry/201909/784395.html.

CHYXX, 2020c. Forecast and price trend analysis of cobalt production, demand, balance between supply and demand in China and the world in 2020. China Industrial Information Network, https://www.chyxx.com/industry/202006/872771.html.

Cobalt Institute, 2020. Cobalt Production Statistics. https://www.cobaltinstitute.org/statistics.html.

Dong, E., Du, H., Gardner, L., 2020. An interactive web-based dashboard to track COVID-19 in real time. Lancet Inf Dis 20(5), 533-534.

Fu, X., Beatty, D.N., Gaustad, G.G., Ceder, G., Roth, R., Kirchain, R.E., Bustamante, M., Babbitt, C., Olivetti, E.A., 2020. Perspectives on Cobalt Supply through 2030 in the Face of Changing Demand. Environ Sci Technol 54(5), 2985-2993.

GGII, 2020. Global shipments of ternary cathode materials. Gao Gong Industrial Research Consulting Co., Ltd.

Huajing Industrial Research Institute, 2020a. Analysis of Chinese manganese dioxide industry chain, production capacity, import and export and development trend. https://www.huaon.com/channel/trend/612778.html.

Huajing Industrial Research Institute, 2020b. Output, pattern and development trend of China anode materials industry in 2019. https://www.huaon.com/channel/trend/614152.html.

IEA, 2020. Global EV Outlook 2020. https://www.iea.org/reports/global-ev-outlook-2020.

IPCC, 2015. Climate Change 2014: Mitigation of Climate Change. Intergovernmental Panel on Climate Change https://www.ipcc.ch/report/ar5/wg3/.

Juda LARGE, 2020. Top 10 global power battery shipments and inventory of the company's production base. http://www.juda.cn/news.

NBSC, 2020. China statistics yearbook 2019. National Bureau of Statistics of China, http://data.stats.gov.cn/easyquery.htm?cn=C01.

Shanghai Xinluo Network Technology, 2020a. China LMO Enterprise Production Ranking in 2019. http://www.iccsino.com/.

Shanghai Xinluo Network Technology, 2020b. China Ternary Cathode Material Enterprise Production Rankings 2019. http://www.iccsino.com/.

Shanghai Xinluo Network Technology, 2020c. The production of lithium iron phosphate is expected to exceed 80,000 tons in 2019. http://www.iccsino.com/.

Sun, X., Hao, H., Liu, Z., Zhao, F., Song, J., 2019. Tracing global cobalt flow: 1995–2015. Resources, Conservation and Recycling 149, 45-55.

Sun, X., Hao, H., Zhao, F., Liu, Z., 2018. Global Lithium Flow 1994-2015: Implications for Improving Resource Efficiency and Security. Environ Sci Technol 52(5), 2827-2834.

UN Comtrade, 2020. Trade data. United Nations Comtrade https://comtrade.un.org/data/.

USGS, 2017a. 2015 Minerals yearbook cobalt. United States Geological Survey,

https://minerals.usgs.gov/minerals/pubs/commodity/cobalt.
USGS, 2017b. 2015 Minerals yearbook nickel. United States Geological Survey, https://minerals.usgs.gov/minerals/pubs/commodity/nickel/.
USGS, 2020. Mineral commodity summaries 2020. United States Geological Survey, https://minerals.usgs.gov/minerals/pubs.
Wood Mackenzie, 2020. Global nickel chemical production will peak in 2027.
